# A Corpus Study of Lexical Bundles Used Differently in Dissertations Abstracts Produced by Chinese and American PhD Students of Linguistics

**DOI:** 10.3389/fpsyg.2022.893773

**Published:** 2022-06-30

**Authors:** Kai Bao, Meihua Liu

**Affiliations:** ^1^College of Foreign Languages, University of Shanghai for Science and Technology, Shanghai, China; ^2^Department of Foreign Languages and Literatures, Tsinghua University, Beijing, China

**Keywords:** lexical bundle, dissertation abstract, corpora, rhetorical move, structure of BUDs, function of BUDs, distribution of BUDs

## Abstract

This study examined lexical bundles (LBs) used differently by Chinese and American PhD students of linguistics in their dissertation abstracts. Two corpora were built, with each having 700 dissertation abstracts produced by Chinese and American PhD students of linguistics, respectively. The study then used lexical analysis software to retrieve frequently used three-word LBs, from which LBs having different frequencies at a significant level across the two corpora were identified and termed as bundles used differently (BUDs). BUDs were then categorized and analyzed manually in terms of structure, function, and distribution in rhetorical moves. The major findings were: (1) 57.14% of the frequently used LBs were BUDs, of which 90.67% had occurrences in both corpora. The BUDs distributed inequivalently across categories and moves, with the text-oriented category and the move of Result having the most BUDs; and (2) BUDs exhibited two major patterns: the Chinese and American students filled different constituents into structurally and functionally similar constructions, and used LBs of dissimilar functions to fulfill the same communicative purposes. These findings indicate that variations in LB use have a high pedagogic value and confirm the need for using corpora to identify and teach core genre-specific vocabularies to second/foreign language learners.

## Introduction

Formulaic language has received increasing attention from linguistics researchers over the past 3 decades. Despite an inconsistency of terminologies, such as constructions (Fillmore, 1988), idiomatic expressions (Titone and Connine, 1999), and LBs (Biber et al., 1999), a consensus has been reached that a substantial proportion of our language in use is comprised by formulaic language (Schmitt and Carter, 2004). Research shows that “stored and retrieved whole” (Wray, 2002, p. 9) multi-word items, an important type of formulaic language, account for as much as 58.60% of English-spoken discourse and 52.30% of written discourse (Erman and Warren, 2000), and are, therefore, “important building blocks of discourse in spoken and written registers” (Biber and Barbieri, 2007, p. 263). Research reveals that learners process formulaic sequences in a considerably shorter reaction time than they process non-formulaic ones in reading (Jiang and Nekrasova, 2007) and speaking (Lin, 2010).

Formulaic language competence is increasingly considered as an indicator of language proficiency (Cortes, 2004). In academic prose, the mastery of discipline-specific sequences demonstrates membership of a discourse community (Segalowitz, 2010; Ädel and Erman, 2012). Formulaic language is particularly important for L2 (second language) learners because “it reduces the learning burden while maximizing communicative ability” (Ellis, 1994, p. 86). Learning formulaic sequences can effectively promote learners’ genre competence by offering ready-made sets of items to work with (Myles, 2004; Coxhead and Byrd, 2007), and allows them to use language with “a single mental effort” (Hunston, 2002, p. 174). Formulaic language, therefore, has high pedagogic value for English for academic purpose (EAP) instruction for L2 learners.

Lexical bundles (LBs), as an important component of formulaic language, are “sequences of words that commonly go together in natural discourse” (Biber and Conrad, 1999, p. 184). The defining features of LBs are non-idiomaticity, structural incompleteness, and frequency-driven identification, with which LB research extends to linguistic items rarely captured with conventional approaches. For instance, *in this dissertation I* is a four-word LB used by American PhD students to present goals of research projects. The sequence, however, is rarely identified by traditional phraseology due to its grammatical incompleteness.

LBs have been widely examined across EAP contexts, including textbooks (e.g., Biber et al., 2004; Liu and Chen, 2020), research papers across different disciplines (e.g., Cortes, 2004; Hyland, 2008a,b), research papers or essays produced by L1 (first language) and L2 English writers (e.g., Chen and Baker, 2010; Pan et al., 2016; Bychkovska and Lee, 2017), and so on. LBs in abstracts have been examined mainly in L1 and L2 English writers’ research articles (RAs) (e.g., Hu and Huang, 2017; Liu and Lu, 2019; Kim and Lee, 2021), whereas those in other genres, such as conference (e.g., Wongwiwat, 2016) and dissertation abstracts (e.g., Lu and Deng, 2019), have been inadequately researched. Dissertations and RAs are distinct genres and use language differently (Hyland, 2008a; El-Dakhs, 2020). Coupled with the rapidly rising number of graduate students who are going to be professionals and need to write in English in different areas in China (e.g., the number of new graduate students was over 1.1 million in 2021, while it was around 0.5 million in 2016), use of LBs in dissertation abstracts, which are the only section required to be written in English as well in most disciplines, deserves research.

In addition, the current LB literature approaches different use of LBs primarily by comparing LBs beyond a frequency cut-off, but rarely by directly examining those having significantly different frequencies across corpora. For instance, Dahunsi and Ewata (2022) compared three-word LBs having 50 or more occurrences in L1 and L2 writers’ texts, showing different structural patterns and colligational characteristics. Their results revealed certain features of LBs used by L1 and L2 writers (e.g., L2 writers used a greater number/proportion of LBs within a certain structure than L1 writers), but did not comprehensively reveal what LBs were used with frequencies different at a significant level by the two groups. It is often assumed that shared bundles in L1 and L2 corpora have often been learned or acquired by L2 learners, while the bundles used (significantly) differently often indicate L2 leaners’ tendency to use the bundles that they have accustomed/trained to and inability to freely employ the bundles frequently used by L1 speakers. Thus, LBs used significantly differently by L1 and L2 learners are more useful to L2 learners in that they represent items to be specifically noticed in L2 learners’ academic writing to enhance its genre-nativeness. Consequently, the present research seeks to examine the LBs used differently by Chinese and American PhD students of linguistics in their dissertation abstracts, hoping to help teach and learn this specific genre more effectively.

## Studies on Lexical Bundles

LBs are “recurrent expressions, regardless of their idiomaticity, and regardless of their structural status” (Biber et al., 1999, p. 990). LBs have transparent meanings interpretable by their individual components (Liu and Chen, 2020), and, therefore, perform categorical discourse functions (Oakey, 2020) and fulfill particular pragmatic purposes (Biber et al., 2004).

LB research often uses pre-defined taxonomies to categorize and analyze LBs in terms of structure, function, as well as communicative purpose. Popular structural (Table 1) and functional taxonomies (Table 2) are proposed by Biber et al. (1999) and Hyland (2008a) for LBs in academic discourse, respectively. In addition, Swales and Feak’s (2009) five-move model (Table 3) has been used in research on LBs in abstracts. LB researchers generally adopt/adapt these models according to the scope and need of their studies.

**TABLE 1:** Biber et al.’s (1999, p. 1014–1015) structural taxonomy.

Structure	Example
Noun phrase + of	*the nature of the*, *a large number of*
Noun phrase + other post-modifier fragment	*the way in which*, *the relationship between*
Prepositional phrase + of	*on the basis of*, *in the context of*
Other prepositional phrase	*on the other hand*, *with respect to the*
Be + noun/adjective phrase	*is due to the*, *is the same as*
Passive verb + prepositional phrase fragment	*is based on the*, *can be found in*
Anticipatory *it* + verb/adjective phrase	*it is important to*, *it should be noted*
(Verb phrase) + *that*-clause fragment	*should be noted that*, *we assume that the*
(Verb/adjective) + *to*-clause fragment	*to be able to*, *are likely to be*
Adverbial clause fragment	*if there is a*, *as can be seen in*
Pronoun/noun phrase + be	*this is not the*, *this is the first*
Other expressions	*than that of the*, *may or may not*

**TABLE 2:** (Hyland, 2008a, p. 49) functional taxonomy.

Category	Examples
**Research-oriented**: help writers to structure their activities and experiences of the real world	
Location: indicate time and place	*at the beginning of*, *at the same time*
Procedure	*the use of the*, *the operation of the*
Quantification	*the magnitude of the*, *a wide range of*
Description	*the structure of the*, *the size of the*
Topic: related to the field of research	*in the Hong Kong*, *the currency board system*
**Text-oriented**: concern with the organization of the text and the meaning of its elements as a message or argument.	
Transition signals: establishing additive or contrastive links between elements	*on the other hand*, *in addition to the*, *in contrast to the*
Resultative signals: mark inferential or causative relations between elements	*as a result of*, *it was found that*, *these results suggest that*
Structuring signals: text-reflexive markers which organize stretches of discourse or direct reader elsewhere in text	*in the present study*, *in the next section*
Framing signals: situate arguments by specifying limiting conditions	*in the case of*, *with respect to the*, *on the basis of*
**Participant-oriented**: focus on the writer or reader of the text.	
Stance features: convey the writer’s attitudes and evaluations	*are likely to be*, *may be due to*, *it is possible that*
Engagement features: address readers directly	*it should be noted that*, *as can be seen*

*The bold font denotes major categories.*

**TABLE 3:** (Swales and Feak, 2009, p. 5) five-move model of abstracts.

Move #	Primary label	Primary function
Move 1	Background	Introducing background/situation
Move 2	Goal	Presenting research/aim
Move 3	Methodology	Stating methodology/materials/subjects/procedures
Move 4	Result	Displaying results/findings
Move 5	Conclusion	Discussing conclusions/implications

Comparative LB research, when at its birth, often compared LBs beyond a frequency cut-off across corpora. For example, Biber et al. (1999) conducted the first LB research and found that LBs in conversation mainly consisted of clause segments, including declarative structures (e.g., *I don’t know what*) and interrogative structures (e.g., *what are your doing*), and that those in academic prose mainly consisted of nominal (e.g., *the end of the*) or prepositional elements (e.g., *on the basis of*). Later, LB studies began to compare LBs frequently used across corpora, featuring different registers (e.g., Biber, 2006; Biber and Barbieri, 2007; Huang, 2018), disciplines (e.g., Cortes, 2004; Hyland, 2008b; Liu and Chen, 2020), genres (e.g., Cortes, 2004; Hyland, 2008a; Gao, 2017), and writer groups (e.g., Nekrasova, 2009; Chen and Baker, 2010; Ädel and Erman, 2012).

Most research on abstracts focuses on LBs in RAs across rhetorical moves (e.g., Omidian et al., 2018; Abdollahpour and Gholami, 2019; Qi and Pan, 2020) and written by L1 and L2 English writers (e.g., Niu, 2014; Hu, 2015; Hu and Huang, 2017), revealing distributional patterns of LBs used differently across corpora. Several recent studies have examined LBs in dissertation abstracts. Lu and Deng (2019) compared LBs in 13,596 and 4,755 dissertation abstracts produced by PhD students of all available disciplines at Tsinghua University and Massachusetts Institute of Technology, respectively. The results showed substantial frequency differences of LBs in structural and functional categories (e.g., the Chinese and American students used 1,640.83 and 786.87 tokens of VP-based LBs per million words, respectively). Similar results were achieved by Lyu and Gee (2020) who compared thesis abstracts written by Chinese and American master students of culture, linguistics, literature, and pedagogy. Li et al. (2020) conducted a bundle-driven analysis to reveal sentence initial LBs in rhetorical moves of dissertation abstracts produced by British PhD students of arts and humanities, social sciences, life sciences, and physics. The study identified a new rhetorical move—structure. Even so, research on LBs in dissertation abstracts is still limited.

Probably because of complexity, little research has specially investigated LBs used with frequencies different at a significant level across corpora, which helps reveal what and how certain LBs are used differently across corpora. Hence, what LBs are used differently for the same functions and communicative purposes is still inadequately researched. This study defines LBs used differently as items whose frequencies are different at a significant level (*p* < 0.05) across corpora, as shown by the log-likelihood (LL) value equal to or greater than 3.84. LL tests perform effectively particularly in comparing the frequencies of low-frequency items across corpora of different sizes (Dunning, 1993; Rayson and Garside, 2000), and are considered “useful for comparing the relative frequency of words or phrases” (Simpson-Vlach and Ellis, 2010, p. 492). Despite its limitation suggested by Bestgen (2017), the LL statistics has been widely used in LB or n-gram studies (e.g., Simpson-Vlach and Ellis, 2010; Ädel and Erman, 2012; Du, 2013; Pan et al., 2016; Bychkovska and Lee, 2017; Hu and Huang, 2017; Hyland and Jiang, 2018, 2021; Lu and Deng, 2019; Jiang and Hyland, 2022).

The LBs used differently by L1 and L2 English writers often indicate L2 learners’ tendency to use the bundles that they have accustomed/trained to and inability to freely employ the bundles frequently used by L1 speakers. Thus, knowledge of differently used bundles is useful to instructors who can use the knowledge to formulate an appropriate pedagogy to help L2 learners use a more variety of LBs in their academic writing and thus enhance its genre-nativeness. English has become “global lingua franca of academia” (Mauranen et al., 2010, p. 183) that “is no one’s first language” (Hyland, 2019, p. 19). Hence, appropriate use of commonly used formulaic English in dissertation abstracts can help promote Chinese PhD students’ readership and academic influence in the international academic community (Hu and Huang, 2021).

All these motivated the present research, which aimed to identify the profiles of LBs used differently at a significant level by Chinese and American PhD students in their linguistics dissertation abstracts and examine how they are used differently by the two groups. And the following two research questions were formulated:

(1) What are the profiles of LBs used differently in dissertation abstracts produced by Chinese and American PhD students of linguistics?
a. What are the forms and frequencies of the LBs used differently in the two corpora?
b. What are the functional and rhetorical move distributions of the LBs used differently in the two corpora?
(2) How do the two groups use different LBs to achieve the same functions and communicative purposes in their dissertation abstracts?
a. How are LBs used for the same functions in the two corpora?
b. How are LBs used for the same communicative purposes in the two corpora?

## Research Design

### Corpora

We first searched for dissertations completed by Chinese PhD students of linguistics between 2000 and 2020 from 13 Chinese universities *via* China National Knowledge Infrastructure, the National Library of China, and relevant university libraries. All the universities were qualified to reward doctoral degrees and tier-1 universities recognized by the Ministry of Education of the People’s Republic of China. The time frame covered a substantial proportion of the genre given that many of the PhD programs in China were founded around 2000. Finally, we gathered 700 dissertation abstracts and established the Chinese University Linguistics Dissertation Abstracts Collection (CUC), which had an overall token of 613,713 and a mean token of 876.73 (SD = 509.44).

To parallel with Chinese universities, high-ranking American universities on 2020 QS World University Rankings by linguistics were selected. Seven hundred dissertations abstracts completed by PhD students of linguistics from these 13 universities in the same time frame were gathered *via* ProQuest and university libraries to establish the American University Linguistics Dissertation Abstracts Collection (AUC). It had an overall token of 247,359 and a mean token of 353.37 (SD = 124.63). The source universities and amounts of the sample texts are presented in the Supplementary Material.

### Data Analyzing Framework

#### Identifying LBs

The present research focused on three-word LBs because they are considerably more frequent than four- and five-word LBs (Hyland, 2008a) and the focus of many existing LB studies (e.g., Hu, 2015, Huang, 2018; Azad and Khiabani, 2018). A consistent selection of length promotes comparability. This study used the WordSmith Tools 8.0 (Scott, 2020) to retrieve three-word LBs based on a cut-off point of 60 occurrences per million words (pmw) in at least 2% (14 texts in CUC and AUC, respectively) of the sample texts. This study adopted a relatively higher frequency cut-off point because the dispersion rate is a more salient parameter in research concerning abstracts that are generally mini texts. In this study, for instance, an adoption of Hyland (2008a,b) 10% dispersion rate entailed 283 occurrences pmw for AUC, and 114 for CUC, which yielded less than 30 items. We, therefore, adopted 2% dispersion cut-off, which was 14 texts and thus 60 occurrences pmw. The cut-off point was consistent with and even stricter than Lu and Deng’s (2019) 20 occurrences and 2.70–9.70‰, and Omidian et al.’s (2018) 20 occurrences and 5.10‰ parameters.

After the retrieval, the overlapping items were combined with the approach proposed by Chen and Baker (2010) to reduce frequency inflation. The LBs underlying complete subsumption where two or more LBs overlapped and one LB subsumed the other(s) were combined (e.g., *as well as* subsumed all occurrences of *well as the* and *well as a* that were, therefore, combined into *as well as*). Those underlying complete overlaps where two three-word LBs were actually one four-word LBs were combined into longer items (e.g., all tokens of *it is argued* and *is argued that* were largely similar and, therefore, were combined into *it is argued that*).

To identify LBs whose frequencies varied at a significant level across CUC and AUC, we used Rayson’s (2016) Effect Size Calculator to yield the LL values. LBs of LL values greater than 3.84 (*p* < 0.05) were recognized as bundles used differently (BUDs). BUDs of a significantly greater frequency in CUC were identified as CUC BUDs and those in AUC as AUC BUDs. We also used the Effect Size Calculator to identify LBs of a significantly greater frequency in a rhetorical move than in a whole text. LBs with LL values greater than 3.84 (*p* < 0.05) were recognized as move-specific bundles and considered a key to constructing a specific move, given their specific occurrences.

#### Categorizing LBs

This study categorized LBs in terms of their structure, function, and distribution in rhetorical moves. The working structural taxonomy, functional taxonomy, and the rhetorical moves, as well as communicative purposes, are provided in the Supplementary Material.

Structurally, we used Biber et al.’s (1999) taxonomy developed specifically for academic prose, and conducted modifications to enhance its relevance to the current genre. For instance, we replaced the *noun + verb phrases + that-clause* with *subject + verb phrases + (that-clause)* to account for a considerable number of AUC items containing first person singular *I*.

Functionally, we used Hyland’s (2008a) functional taxonomy developed specifically for LBs in RAs and dissertations, and added new sub-categories, including relationship signals (LBs denoting relationships between entities, such as *is consistent with*), objective signals (LBs denoting objectives, such as *in order to*), and other bundles. We also promoted inferential and causative signals to sub-categories within the text-oriented category. To reduce subjectivity on functional categorization, a PhD student of linguistics was employed to categorize LBs independently with the researcher. Our Cohen’s Kappa coefficient was 0.889 (*k* > 0.80), indicating an excellent strength of inter-coder agreement (Landis and Koch, 1977). For LBs whose functions were disagreed upon, the two coders went through and discussed the items until they reached complete consensus.

With regard to rhetorical moves, we used Swales and Feak’s (2009) five-move model and followed Li et al. (2020) to add the move of structure that specifically outlined dissertation structures found in many CUC and AUC texts in the present study. Based on the model, the two researchers identified moves and their communicative purposes independently, with a Kappa coefficient of 0.813. We then went through and discussed the disagreed-upon items until we reached complete consensus.

#### Analyzing LBs

As discussed, we identified LBs frequently used in the two corpora *via* WordSmith Tools 8.0 (Scott, 2020), with a cut-off point of 60 occurrences pmw in at least 2% of the sample texts. We then identified BUDs from the LBs frequently used by their LL value (LL value ≥ 3.84, *p* < 0.05), with Rayson’s (2016) Effect Size Calculator.

To reveal how LBs were used differently in the two corpora, we examined the structures of BUDs in the same functional categories to determine whether the BUDs of the same functions resulted from different structures. Instead of analyzing tokens of BUDs serving different functions in rhetorical moves, we analyzed the functions of BUDs identified as move-specific bundles, serving the same communicative purposes in rhetorical moves, because: (1) rhetorical moves in dissertation abstracts are often more informative than those in RAs in that dissertation abstracts are considerably longer and contain LBs, serving various communicative purposes; and (2) move-specific bundles are key items used to construct moves whose occurrences have move-specific features.

## Results

### Profiles of Bundles Used Differently in the Two Corpora

#### Forms and Frequencies

According to the cut-off point, 274 bundle types with 20,154 tokens and 195 types with 5,798 tokens were identified in CUC and AUC, respectively, as shown in Table 4 (full lists are in the Supplementary Material). In terms of token frequency, the Chinese students used a substantially greater number of LBs than their American counterparts (LL value = 543.41, *p* < 0.0001). Regarding bundle forms, only 101 bundle types were shared in the two corpora, suggesting that 63.14% of the bundle types frequent in CUC were not frequent in AUC, and that 48.21% of the bundle types frequent in AUC were not frequent in CUC. These results indicated substantial variations across CUC and AUC with regard to both frequencies and forms. The LBs frequently used by the Chinese and American students were, therefore, considerably dissimilar.

**TABLE 4:** Number of frequently used LBs and BUDs.

Corpus	LBs		BUDs		BUD occurrences in CUC and AUC	
	Type	Token	Type	Percentage in LBs	Type	Percentage in BUDs
CUC	274	20,154	175	63.87%	157	89.71%
AUC	195	5,798	93	47.69%	86	92.47%

Of the frequently used LBs, 268 BUDs that represented the LBs used differently by the two groups were identified. Of the 268 BUDs, 175 were CUC BUDs, indicating that 63.87% of the LBs frequently used in CUC had significantly greater frequencies than those in AUC; 93 were AUC BUDs, indicating that 47.69% of those in AUC had significantly greater frequencies than those in CUC. It should be noted that 157 of the 175 (89.71%) CUC BUDs had occurrences in both collections, and the proportion was 86 of 93 (92.47%) for AUC BUDs. These results showed that the Chinese and American PhD students of linguistics might draw on a quite similar formulaic language resource but with substantially dissimilar frequencies.

#### Distribution in Functional Categories and Rhetorical Moves

As shown in Table 5, the greatest number of BUDs occurred in the text-oriented category, followed by the research- and participant-oriented categories, and description bundles had the largest number of BUDs, followed by framing and structuring signals. It should be noted that inferential signals had 15 AUC, yet only three CUC BUDs, and that inferential and causative signals were the only two categories that had more AUC than CUC BUDs. Meanwhile, topic bundles had 14 CUC BUDs but no AUC BUDs; transition signals had seven CUC BUDs but only one AUC BUD. These results revealed an inequivalent distribution of BUDs across all the three functional categories and their 15 subcategories.

**TABLE 5:** Functional distribution of BUDs.

Category	CUC BUDs		AUC BUDs		Total	
	Type	Percentage	Type	Percentage	Type	Percentage
**Research-oriented**	**71**	**40.57%**	**31**	**33.33%**	102	**38.06%**
Location	3	1.71%	1	1.08%	4	1.49%
Procedure	14	8%	6	6.45%	20	7.46%
Quantification	8	4.57%	8	8.60%	16	5.97%
Description	32	18.29%	16	17.20%	48	17.91%
Topic	14	8%	0	0	14	5.22%
**Text-oriented**	**94**	**53.71%**	**59**	**63.44%**	153	**57.09%**
Transition signals	7	4%	1	1.08%	8	2.99%
Inferential signals	3	1.71%	15	16.13%	18	6.72%
Causative signals	8	4.57%	10	10.75%	18	6.72%
Structuring signals	23	13.14%	11	11.83%	34	12.69%
Framing signals	23	13.14%	12	12.90%	35	13.06%
Relationship signals	16	9.14%	5	5.38%	21	7.84%
Objective signals	14	8%	5	5.38%	19	7.09%
**Participant-oriented**	**10**	**5.71%**	**2**	**2.15%**	12	**4.48%**
Stance features	5	2.86%	1	1.08%	6	2.24%
Engagement features	1	0.57%	1	1.08%	2	0.75%
Other functions	4	2.29%	1	1.08%	5	1.87%
Total	175	100%	93	100%	268	100%

*The bold font denotes major categories and is not counted in the total number.*

Table 6 shows that the greatest number of BUD tokens occurred within the move of Result, followed by moves of Methodology and Goal. These results suggested that the Chinese and American students used substantially different LBs to present results, designs, and purposes of their research projects. It should be noted that the moves of Result and Goal were the only two rhetorical moves that had a greater proportion of AUC than CUC BUDs, while the move of Background had 16.16% of CUC yet only 7.27% of AUC BUDs. These findings indicated the Chinese students’ heavy reliance on formulaic language in composing their dissertation abstracts.

**TABLE 6:** Rhetorical move distribution of BUDs.

Move	CUC BUDs		AUC BUDs	
	Token	Percentage	Token	Percentage
Background	2,203	16.16%	216	7.27%
Goal	1,714	12.57%	623	20.97%
Methodology	2,735	20.06%	560	18.85%
Result	4,777	35.04%	1,330	44.77%
Conclusion	1,825	13.39%	166	5.59%
Structure	378	2.77%	76	2.56%
Total	13,632	100%	2,971	100%

### Analyses of BUDs in the Two Corpora

#### BUDs Within Functional Categories

**FIGURE 1:**
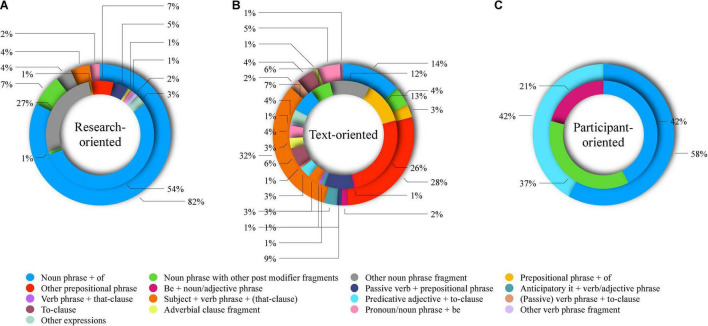
Structural distribution of BUDs in functional major categories (an inner circle: CUC BUDs; an outer circle: AUC BUDs; **(A)** research-oriented; **(B)** text-oriented; and **(C)** participant-oriented).

Figure 1 presents the structural distribution of BUDs in the three major functional categories. As shown in Figure 1A, the CUC BUDs fell into ten subcategories, whereas the AUC BUDs fell into five categories. Four structural categories, including *noun phrase + of*, *noun phrase with other post modifier fragments*, *other noun phrase fragment*, and *pronoun/noun phrase + be*, contained both CUC and AUC BUDs and accounted for 84.13% of all BUD tokens within the research-oriented category. The structural categories specific to AUC and CUC BUDs, including *subject + verb phrase +* (*that-clause*) and *other prepositional phrase*, etc., only accounted for 15.87% of all BUD tokens within this category. Figure 1B show that the CUC BUDs fell into all the 17 subcategories and the AUC BUDs fell into 13 subcategories, which accounted for 84.02% of BUDs within the text-oriented category. Figure 1C shows that the participant-oriented category had three LB types, and that only one structural category, *noun phrase + of*, was shared by CUC and AUC BUDs within this category and accounted for 45.70% of BUDs. These results suggested that BUDs in the two corpora fell into substantially similar structural categories, meaning that what led to the two groups’ different use of LBs with respect to structure was mainly different constituents filled in the functionally and structurally similar constructions, but not different structures used to construct LBs of the same functions.

We then analyzed the constituents filled into the same structures of BUDs within the same functional categories. Table 7 shows five typical pairs of CUC and AUC BUDs, which had the same structures and functions. For example, within location bundles, the Chinese students were more likely to use (*in*) *the process of*, whereas the American students used (*over*) *the course of* to refer to a time period. In Ex. 1 and Ex. 2, both BUDs collocated with *years* in similar contexts.

**TABLE 7:** Example BUDs with different constituents in constructions.

Function	Structure	BUDs
Location bundle	Noun phrase + of	CUC: *the process of* AUC: *the course of*
Description bundle	Noun phrase + of	CUC: *the meaning of* AUC: *the semantics of*
Inferential signal	Subject + verb phrase + that-clause	CUC: *we find that* AUC: *I show that*
Causative signal	Noun phrase + of	CUC: *the findings of* AUC: *the results of*
Structuring signal	Other prepositional phrase	CUC: *in this study* AUC: *in this dissertation*

Ex. 1. In **the process of** 30 years from 1990 to the present, …. (CUC).

Ex. 2. The fieldwork for this study was conducted over **the course of** 7 years. (AUC).

Likewise, within description bundles, while the Chinese students tended to refer to the meaning of lexical items by *the meaning of*, their American counterparts more often used *the semantics of*. The two BUDs usually occurred in the same contexts, as shown in Ex. 3 and 4. The same pattern was identified with many other pairs of BUDs within description bundles commonly used by the Chinese and American students, respectively, including *features of the* and *properties of the*, *characteristics of the* and *the nature of*, and *the framework of* and *a model of*.

Ex. 3. …a “semantic mismatch” between **the meaning of** the lexical items… (CUC).

Ex. 4. …a single underlying difference in **the semantics of** these lexical items… (AUC).

Analyses showed that, within inferential signals, the Chinese students used more *find* as in *it is found*, but the American students used more *argue* and *show* as in *it is argued/shown* in *anticipatory it + verb/adjective phrase* structure. These results indicated the Chinese students’ particular and even exclusive preference for using *find* to report research results. In contrast, the American students used a much wider range of verbs, including *argue*, *show*, *propose*, *suggest*, *indicate*, *demonstrate*, and *propose*, whose occurrences in CUC inferential signals were all significantly fewer. Despite the semantic differences of these verbs, *find* might be the first and most prototypical reporting verb learned and used by many Chinese students who thus felt more confident with the verb (Li et al., 2018). Within inferential signals of *subject + verb phrase +* (*that-clause*) structure, the Chinese students primarily used *we find that* and strictly avoided using LBs containing the first person singular *I* to reduce authorial stance, while these bundles were the most common inferential signals used by the American students. Table 8 shows a total of 10 AUC LBs containing authorial *I*, which were all AUC BUDs. These items had 1,827 tokens pmw in AUC, yet only 21 tokens in CUC. They accounted for 31.51% of the total AUC bundle tokens and were, therefore, the most important bundle group in AUC in this regard. The tokens of the most common *I*-bundle *I argue that* alone made up 3.24% of all bundle tokens in AUC. Functionally, the bundles fell into inferential signals and procedure bundles that indicated writers’ intellectual aspects in research. Meanwhile, 9 of the 10 items were move-specific bundles and thus a key to the construction of specific rhetorical moves, including moves of AUC Result and Methodology. The *I*-bundles were, therefore, crucially important to the American students but strictly avoided by the Chinese students in the *subject + verb phrase +* (*that-clause*) structure.

**TABLE 8:** *I*-bundles.

*I*-bundle	Token frequency		Function	Move (communicative purpose)
	AUC	CUC		
*I argue that*	188	8	Inferential signal	Result (mark the report of results)
*I show that*	96	0	Inferential signal	Result (mark the report of results)
*I propose that*	40	2	Inferential signal	Result (mark the report of results)
*I demonstrate that*	22	0	Inferential signal	Result (mark the report of results)
*I focus on*	19	0	Procedure bundle	Methodology (display research acts)
*I examine the*	19	0	Procedure bundle	Methodology (display research acts)
*I propose a*	20	2	Inferential signal	NA
*I argue for*	18	1	Inferential signal	Result (mark the report of results)
*I suggest that*	15	0	Inferential signal	Result (mark the report of results)
*I show that*	15	0	Inferential signal	Result (mark the report of results)

Meanwhile, the Chinese students used significantly more structuring signals (LL value = 46.19, *p* < 0.0001), such as the CUC BUD *the present study*, as sentence subjects. The subjects of move-specific bundles were primarily *this dissertation* as in *this dissertation examines* in AUC Goal and *the dissertation* as in *the dissertation is* in Structure. This finding showed the American students’ more flexible use of subjects to implicitly mark move boundaries and their attempts to highlight individual contributions to the design and results of their projects.

Within structuring signals, the American students consistently used *dissertation* as in *in this dissertation*, whereas the Chinese students used more often *study* as in *in this study*, *research* as *the present research*, and *dissertation* as in *the present dissertation* to refer to doctoral research. Many BUDs in this category exhibited this feature, including AUC BUDs *of the dissertation*, *this dissertation presents*, as well as *this dissertation is*, and the CUC BUDs *in this research*, *present study has*, and *this study is*. The difference might reflect the Chinese students’ relative unfamiliarity with and, therefore, reluctance to use *dissertation* that specifically denoted doctoral research. The same pattern was identified within causative signals where the Chinese students tended to use *finding*, while the American students primarily relied on *result*, which was reflected in the CUC BUDs *the findings of* and *findings of the*, and the AUC BUD *the results of*. The Chinese students might use *findings* to emphasize their active efforts to *find* facts, whose subtle meaning could not be communicated by *result*. These findings indicate the two writer groups’ different choices of words within the same structures.

#### BUDs Within Rhetorical Moves

**FIGURE 2:**
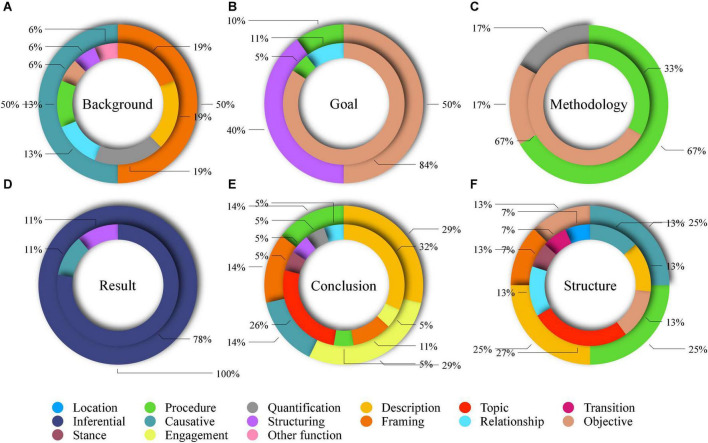
Functional distribution of move-specific bundles in several communicative purposes (an inner circle: CUC; an outer circle: AUC; **(A)** introducing previous studies; **(B)** presenting purposes of research; **(C)** displaying research acts; **(D)** reporting results of research; **(E)** demonstrating beneficiaries of research; and **(F)** stating contents of chapters).

Figure 2 presents the functional distribution of move-specific bundles in the communicative purposes that had most LBs in each of its six sub-moves. The communicative purposes included introducing previous studies (A), presenting purposes of research (B), displaying research acts (C), reporting results of research (D), demonstrating beneficiaries of research (E), and stating contents of chapters (F).

In Background, both groups used most LBs to achieve the communicative purpose of introducing previous studies where 11 BUDs were identified. As shown in Figure 2A, the Chinese students used a much wider functional variety of LBs that included description bundles (e.g., *the previous studies*), quantification bundles (e.g., *most of the*), framing signals (e.g., *the field of*), and procedure bundles (e.g., *the research on*). The American students only used framing signals (e.g., *the field of*) and causative signals (e.g., *it has been*). Similarly, to emphasize the importance of their research, the Chinese students used quantification bundles (e.g., *a lot of*), stance feature bundles (e.g., *the most important*), and transition signals (e.g., *is not only*), whereas the American students only used quantification bundles (e.g., *one of the*).

Figure 2B indicates both groups’ marked reliance on objective signals to present research purposes in Goal. In CUC, 84.21% of the move-specific bundles were objective signals, which were 50% for AUC. This difference indicated the Chinese students’ heavier reliance on objective signals to present research aims. Instead, the American students showed marked reliance on structuring signals that made up 40% of the move-specific bundles, which were rarely used by the Chinese students. Ex. 5 and 6 show the two groups’ typical use of objective and structuring signals in this regard, which shows that the American students’ use of structuring signals commonly followed the pattern *this dissertation + investigates*/*examines*/*presents*/*explores*, while the Chinese students primarily relied on objective signals, such as *study aims to* that contained the verb *aim*, communicating a more explicit meaning of purposes.

Ex. 5. **This dissertation explores** the role of affect in sociolinguistic style. (AUC GOAL).

Ex. 6. The present **study aims to** explore the use of anaphora. (CUC GOAL).

The Chinese students’ greater reliance on objective signals was also identified in displaying research acts in Methodology. Figure 2C shows that 66.67% of the CUC move-specific bundles fell into objective signals, which were only 16.67% for AUC. The American students used 66.67% of the items as procedure bundles that only accounted for 33.33% in CUC. The procedure bundles used by the American students commonly followed the pattern *I + examine/focus on the* in an active voice (Ex. 7). In comparison, objective signals, such as *to analyze the*, which fell into *to-clause* structure were commonly used by the Chinese students in a passive voice (Ex. 8). In addition, in description of research design in Methodology, the CUC items consisted of framing signals (55.56%), procedure bundles (22.22%), and quantification bundles (22.22%), while the AUC items fell equivalently into location bundles, framing signals, and quantification bundles. The Chinese students, therefore, used considerably more framing signals for the purpose. Ex. 9 is a series of CUC sentences that all began with a framing signal that specified the conditions underlying arguments. Such intense use of framing signals was not identified in any move of AUC.

Ex. 7. **I examine the** basic syntax of English comparatives and readers’ expectations for…. (AUC).

Ex. 8. The Paired Sample *t*-test is conducted **to analyze the** tendency regarding…

Ex. 9. **In terms of** mode, we have…; **in terms of** motive, we have…; **in terms of** addressees, the offensive addressing…; **in terms of** degree, we have… (CUC).

Figure 2D reveals both groups’ marked reliance on inferential signals to signal the report of research results. It shows that 100% of the AUC move-specific bundles were inferential signals, which were 77.78% of the CUC items. In addition to objective signals, the CUC items included structuring and causative signals that could be a specific discourse signal used by the Chinese students for the purpose. The LL values of the CUC structuring signal *are as follows*, and causative signals *the major findings* were 34.05 and 17.73, respectively, but that of the inferential signal *we find that* was 13.32. The Chinese students specifically used the combination of *the major findings* and *are as follows* to signal the report of research results (Ex. 10). In comparison, the American students commonly used inferential signals in the pattern *I + argue/show/propose/demonstrate/suggest that*, which could highlight writers’ individual contributions more explicitly (Ex. 11).

Ex. 10. **The major findings** of the present research **are as follows:** 1…2…3… (CUC RESULTS).

Ex. 11. **I show that** these two constructions again mirror the situation…: **I propose that** the two constructions contribute the same semantic pieces… (AUC RESULTS).

Regarding Conclusion, Figure 2E reveals that, despite the two groups’ similar use of description bundles (e.g., *the study of*) to point out the beneficiaries of their research, they generally used different categories as well as proportions of move-specific bundles for the purpose. It shows that 26.32% of the CUC items were topic bundles (e.g., *second language acquisition*) that specified the beneficiary field, which was not identified in AUC. The American students specifically used engagement feature bundles (e.g., *our understanding of*) to build relevance between readers and their research outcomes (Ex. 12). Using participant-oriented bundles for the purpose as well, the Chinese students used the stance feature bundle *better understanding of* that also fell into *noun phrase + of* structure (Ex. 13). To mark the discussion of implications, the Chinese students used a combination of causative signals, structuring signals (Ex. 14), and procedure bundles, different from the American students’ sole use of causative signals (Ex. 15).

Ex. 12. My work contributes to **our understanding of** how power… (AUC CONCLUSION).

Ex. 13. It is hoped that this study can contribute to a **better understanding of**… (CUC CONCLUSION).

Ex. 14. The present study also contributes to the pragmatic field **in the following** aspects. (1)…(2)…(3)… (CUC CONCLUSION).

Ex. 15. **The implications of** this research extend to theories of discourse and reference. (AUC CONCLUSION).

Figure 2F shows the Chinese students’ specific use of topic bundles, relationship signals, stance feature bundles, transition signals, as well as location bundles, and the American students’ specific use of procedure bundles and framing signals to demonstrate the contents of dissertation chapters in Structure. Topic bundles accounted for 26.67% of the CUC items but none of the AUC items, which indicates the Chinese students’ more recurrent mention of research subjects.

## Discussion

### Profiles of BUDs in the Two Corpora

This study revealed that the Chinese students produced significantly more LBs than American students, consistent with the finding in several previous studies that a considerably greater number of English LBs were used by L1 Chinese than L1 English writers (Guan and Zheng, 2005; Hyland, 2008a; Pang, 2009; Lou, 2010; Wei and Lei, 2011; Xu, 2012; Pan et al., 2016; Gao, 2017; Lyu and Gee, 2020). A similar finding is achieved with L1 Spanish speakers (Pérez-Llantada, 2014), L1 Iranian speakers (Jalali et al., 2008), and L1 Turkish speakers (Güngör and Uysal, 2016). These findings generally indicate L2 English writers’ greater reliance on formulaic language to construct academic articles, as discussed in Hyland (2008a) and Paquot and Granger (2012). Hyland (2008a) argued that the considerably higher LB token frequency in L2 writers’ academic writing primarily resulted from apprentice writers’ heavier reliance on prefabricated items in the development of their arguments. Likewise, Paquot and Granger (2012, p. 139) argued that “less-proficient learners seem to be more reliant on lexical bundles.” This is largely because L1 writers normally have a larger repertoire of formulaic language and thus do not have to stick to a smaller variety of high-frequency items, while L2 writers often have a smaller repertoire and have to use high-frequency LBs more recurrently.

In addition, the difference could be partially explained by the generic features of our corpora. Dissertation abstracts are a high-stake genre, concerning both research manifestation and degree fulfillment. While research manifestation is mainly for international readers, degree fulfillment needs to be approved by a dissertation supervision committee whose members are primarily Chinese professors. To cope with the complicated and important writing task in L2, Chinese students may tend to use language sequences with which they feel confident and specify situations of arguments more carefully, especially in a considerably longer text.

The present study also found that, although the LBs frequently used by the two groups were substantially different with respect to forms and frequencies, about 90% of the BUDs had occurrences in both collections. In addition, BUDs distributed inequivalently across functional categories and rhetorical moves. This finding was new since most existing LB studies focus mainly on frequently used LBs (e.g., Hyland, 2008a; Lu and Deng, 2019; Lyu and Gee, 2020), but rarely on LBs of significantly different frequencies across corpora. By focusing on BUDs, this study revealed that the Chinese and American students drew on a highly similar resource of formulaic sequences but selected items with considerably different frequencies. Nevertheless, these findings need to be confirmed in more similar research.

### Patterns of BUDs in the Two Corpora

This study showed that the Chinese and American students filled in different constituents into the structurally and functionally similar constructions, indicating the two groups’ different choices of lexical bundles.

Within inferential signals of *subject + verb phrase +* (*that-clause*) structure, the Chinese students primarily used *we find that* and strictly avoided using LBs containing the first person singular *I* to reduce authorial stance, but these bundles were the most common inferential signals used by the American students. The first person plural refers to the research work or the author in CUC, which ought to be singular because dissertations are individual works. The Chinese students’ use of *we* probably resulted from their avoidance of authorial *I* or a “misunderstanding of rhetorical conventions” (Li et al., 2018, p. 42). Scholars in support of the avoidance of personal pronouns in academic prose argue that an unrestrained use of authorial *I* reduces the objective expression of ideas (Arnaudet and Barrett, 1984; Kirsch, 1994; Spencer and Arbon, 1996). The first person viewpoint, however, has become more acceptable in academia over recent years. Ivanic (1998) argued that the first person singular in academic writing is a powerful tool for self-representation and the construction of authorial identity. Similarly, Kuo (1999) and Hyland (2001) argued that a proper use of authorial *I* could promote the emphasis on the authors’ individual contribution to a research field. Notably, Section 4.16 of the newest APA style (American Psychological Association, 2020) as well as Section 5.40 of the newest Chicago Manual of Style (The University of Chicago Press Editorial Staff, 2017) both encourage the use of first person pronouns to describe research work and personal reactions.

The study also revealed that the *I*-bundles were the most important AUC bundle group in terms of token frequency but were strictly avoided in CUC. The Chinese students’ avoidance of using *I* might result from the conventional EAP protocol that requires writers to conceal the authorial aspects in favor of readers’ closer focus on the substance of writing (Arnaudet and Barrett, 1984; Kirsch, 1994; Spencer and Arbon, 1996). Since dissertations might be the most important piece of writing in PhD students’ lives (Hyland, 2008a), Chinese students are advised to use these bundles by substituting authorial *I* with names of research, chapters, or sections, such as *this dissertation/study/chapter/section argues that* for *I argue that*. The sequence is able to help Chinese students promote an authorial voice without breaking the protocol.

This study also showed that the Chinese and American students used LBs of dissimilar functions to fulfill the same communicative purposes. This might be related to the generic features of CUC. For instance, the causative signal *the major findings* and the structuring signal *are as follows* can be more effective discourse markers than inferential signals, such as *I show that* to signal a considerably longer report of results, as discussed in Allen (2009) and Qin (2014). The more frequent use of objective signals like *study aims to* in presenting research aims can be a more familiar discourse signal for Chinese professors, especially in a substantially longer text, so are structuring signals like *the present study* that marks implications in Conclusion, as discussed in Cortes (2004).

## Conclusion and Implications

This study examined three-word LBs in 1,400 dissertation abstracts written by Chinese and American PhD students of linguistics and focused on BUDs, which were LBs used with significantly different frequencies by the two groups. The study had two major findings. First, most BUDs had occurrences in both corpora. The BUDs distributed inequivalently across functional categories and rhetorical moves, with the text-oriented category and move of Result having the most BUDs. Second, the two groups used similar structures to construct LBs of the same functions but filled different constituents into structurally and functionally similar constructions. They also used dissimilar functions for the same communicative purposes in rhetorical moves. Many BUDs related to the considerably lengthier texts in CUC than in AUC. It should be noted that the Chinese students strictly avoided filling first person singular *I* in *subject + verb phrase +* (*that-clause*) structure to reduce authorial stance, while these bundles were the most common inferential signals used by the American students to report results of their research.

These findings reveal not only general profiles of BUDs in dissertation abstracts produced by Chinese and American PhD students of linguistics but also how they used BUDs to fulfill the same functions and communicative purposes. The general profiles indicate a similar lexical resource from which the Chinese and American students picked items with substantial different frequencies, categories, and moves of greater number of BUDs and thus greater variations. Hence, the bundle lists generated by this study can be used in teaching. An instructor can also use lexical analysis tools to identify bundles used differently by Chinese students compared with their American counterparts, and then formulate a bundle list. The instructor can then focus on reducing certain items and choosing a variety of appropriate items within a certain functional category or rhetorical move from her or his students’ writing in order to enhance genre-nativeness. For instance, an instructor can ask his/her students to use more (*over*) *the course of* instead of (*in*) *the process of* to denote a time period, *the extent to which* to replace *the degree of*, and *specifically* to replace *that is the* in the same context. This can help reduce both CUC and AUC BUDs whose frequencies offset each other. Meanwhile, because of the availability of various corpora, students are encouraged to discover patterns of and differences in the use of LBs by different learner groups to improve their own writing.

Additionally, our finding of specific patterns underlying different uses of LBs shows that not all BUDs ought to be specifically noticed. The BUDs that relate to generic features (e.g., of *in the following* vs. *I show that*, the former marked a considerably lengthier move of Result in CUC), such as substantially different word counts and potentially different discoursal expectations toward the genre, should not be the focus of instruction. Instead, the BUDs that relate to different linguistic choices in the same structure, function, as well as context should be paid closer attention to (e.g., both the *process* of vs. the *course of* denote a period of time). Instructors are also advised to teach the AUC BUDs containing authorial *I* to Chinese students by asking them to replace *I* with the name of study, chapter, or section (e.g., *I argue that* taught as *this dissertation argues that*). The revised sequence emphasizes individual contributions and, at the same time, adapts to the traditional EAP convention that discourages using authorial *I*. The instructor can also measure learning progress by using lexical analysis tools to compare LBs in L2 learners’ writing against those produced by native learners of English. As discussed in Kazemi et al. (2014), bundle-based instruction raises learners’ awareness of formulaic language and develops their English writing skills.

This study confirms the need for using corpus linguistics to identify and teach core genre-specific vocabularies to L2 learners. Even so, the research has certain limitations. The biggest limitation is that it does not analyze the multifunctionality of LBs because of the subjective nature of functional categorization and the complexity of contexts. A study in a specific context may grant us a closer understanding of LB functions. Secondly, the study was based on corpora of texts with different average lengths that might affect the use of LBs (Pan et al., 2020). Future studies can use corpora of texts with a similar average length to complement our investigation. Thirdly, the present research only investigated dissertation abstracts produced by Chinese and American PhD students of linguistics. A comparative study between different disciplines will enrich the literature on dissertation abstracts. Such studies can help compile core genre-specific bundle lists that include structural, functional, and rhetorical move distributions to support instruction and research on EAP vocabularies. In addition, future studies can apply psycholinguistic experiments to determine the effects of and causes for BUDs. L1 and L2 English writers’ reactions to BUDs can help instructors develop tailored teaching resources, techniques, and strategies.

## Data Availability Statement

The raw data supporting the conclusions of this article will be made available by the authors, without undue reservation.

## Ethics Statement

Ethical review and approval was not required for the study on human participants in accordance with the local legislation and institutional requirements. Written informed consent for participation was not required for this study in accordance with the national legislation and the institutional requirements.

## Author Contributions

KB and ML designed the research, analyzed the data, and wrote the manuscript. Both authors contributed to the article and approved the submitted version.

## Conflict of Interest

The authors declare that the research was conducted in the absence of any commercial or financial relationships that could be construed as a potential conflict of interest.

## Publisher’s Note

All claims expressed in this article are solely those of the authors and do not necessarily represent those of their affiliated organizations, or those of the publisher, the editors and the reviewers. Any product that may be evaluated in this article, or claim that may be made by its manufacturer, is not guaranteed or endorsed by the publisher.
