# Supplementary material for: A Corpus Study of Lexical Bundles Used Differently in Dissertations Abstracts Produced by Chinese and American PhD Students of Linguistics
Source: Front Psychol. 2022 Jun 30;13:893773. doi: 10.3389/fpsyg.2022.893773 (PMC9280359; doi:10.3389/fpsyg.2022.893773)
Supplement: Supplementary file 1 [file Data_Sheet_1.docx]

Supplementary Material

# Frequent lexical bundles

Table 1 and 2 show the frequent lexical bundles in CUC and AUC respectively, and the LB structure, function, as well as distribution in the six rhetorical moves.

Table 1. Frequent CUC lexical bundles

| Lexical bundle | Token | Text | Structure | Function | Token in rhetorical moves | | | | | |
| --- | --- | --- | --- | --- | --- | --- | --- | --- | --- | --- |
|  |  |  |  |  | B | G | M | R | C | S |
| *the present study* | 585 | 237 | other noun phrase | structuring signal | 28 | 132 | 142 | 80 | 186 | 17 |
| *in terms of* | 486 | 265 | prepositional phrase + of | framing signal | 68 | 30 | 112 | 226 | 43 | 7 |
| *as well as* | 398 | 239 | other expressions | transition signal | 65 | 30 | 91 | 137 | 54 | 21 |
| *based on the* | 379 | 274 | passive verb + prepositional phrase | framing signal | 34 | 46 | 124 | 121 | 43 | 11 |
| *English and Chinese* | 348 | 88 | other noun phrase | topic bundle | 31 | 50 | 62 | 162 | 24 | 19 |
| *Chinese EFL learners* | 284 | 63 | other noun phrase | topic bundle | 13 | 82 | 55 | 101 | 33 | 0 |
| *the study of* | 280 | 138 | noun phrase + of | description bundle | 122 | 22 | 51 | 28 | 48 | 9 |
| *the use of* | 265 | 126 | noun phrase + of | description bundle | 38 | 21 | 27 | 155 | 24 | 0 |
| *in order to* | 259 | 176 | other prepositional phrase | objective signal | 39 | 28 | 93 | 68 | 29 | 2 |
| *the process of* | 247 | 144 | noun phrase + of | location bundle | 57 | 16 | 37 | 101 | 31 | 5 |
| *on the basis of* | 217 | 161 | prepositional phrase + of | framing signal | 13 | 31 | 98 | 57 | 16 | 2 |
| *Chinese and English* | 209 | 67 | other noun phrase | topic bundle | 15 | 34 | 49 | 90 | 19 | 2 |
| *the development of* | 181 | 109 | noun phrase + of | description bundle | 42 | 15 | 28 | 51 | 42 | 3 |
| *one of the* | 180 | 136 | noun phrase + of | quantification bundle | 95 | 4 | 28 | 39 | 12 | 2 |
| *from the perspective of* | 176 | 125 | prepositional phrase + of | framing signal | 41 | 43 | 30 | 34 | 23 | 5 |
| *the relationship between* | 168 | 111 | noun phrase + other post modifier | relationship bundle | 44 | 27 | 32 | 35 | 24 | 6 |
| *in this study* | 151 | 112 | other prepositional phrase | structuring signal | 4 | 19 | 63 | 39 | 25 | 1 |
| *according to the* | 144 | 115 | predicative adjective + to-clause | framing signal | 31 | 1 | 47 | 58 | 3 | 4 |
| *what are the* | 141 | 76 | pronoun phrase/noun phrase + be | other bundle | 5 | 125 | 11 | 0 | 0 | 0 |
| *the analysis of* | 138 | 104 | noun phrase + of | procedure bundle | 16 | 11 | 40 | 46 | 22 | 3 |
| *on the other* | 138 | 108 | other prepositional phrase | transition signal | 27 | 3 | 20 | 65 | 23 | 0 |
| *the present research* | 131 | 63 | other noun phrase | structuring signal | 5 | 29 | 27 | 35 | 31 | 4 |
| *of this study* | 130 | 100 | other prepositional phrase | structuring signal | 1 | 18 | 24 | 30 | 49 | 8 |
| *to explore the* | 128 | 109 | to-clause | objective signal | 19 | 49 | 37 | 7 | 13 | 3 |
| *so as to* | 128 | 90 | adverbial clause fragment | objective signal | 18 | 19 | 25 | 41 | 25 | 0 |
| *in this dissertation* | 128 | 84 | other prepositional phrase | structuring signal | 7 | 10 | 45 | 33 | 22 | 11 |
| *between the two* | 127 | 82 | other prepositional phrase | relationship bundle | 21 | 6 | 19 | 74 | 4 | 3 |
| *of the study* | 124 | 81 | other prepositional phrase | structuring signal | 9 | 11 | 40 | 29 | 21 | 14 |
| *the effects of* | 116 | 63 | noun phrase + of | causative signal | 18 | 30 | 32 | 23 | 13 | 0 |
| *of the Chinese* | 114 | 55 | other prepositional phrase | topic bundle | 17 | 10 | 24 | 48 | 13 | 2 |
| *the influence of* | 112 | 72 | noun phrase + of | causative signal | 17 | 11 | 28 | 45 | 7 | 4 |
| *part of the* | 110 | 74 | noun phrase + of | description bundle | 17 | 0 | 33 | 46 | 6 | 8 |
| *in the study* | 109 | 84 | other prepositional phrase | structuring signal | 27 | 7 | 49 | 14 | 9 | 3 |
| *due to the* | 108 | 93 | predicative adjective + to-clause | causative signal | 23 | 5 | 14 | 52 | 14 | 0 |
| *of the two* | 107 | 77 | other prepositional phrase | framing signal | 17 | 10 | 13 | 56 | 11 | 0 |
| *the meaning of* | 104 | 66 | noun phrase + of | description bundle | 27 | 1 | 22 | 49 | 5 | 0 |
| *the role of* | 102 | 67 | noun phrase + of | causative signal | 26 | 15 | 19 | 30 | 11 | 1 |
| *the results of* | 101 | 75 | noun phrase + of | causative signal | 4 | 1 | 17 | 56 | 22 | 1 |
| *study of the* | 101 | 75 | noun phrase + of | description bundle | 16 | 17 | 27 | 20 | 19 | 2 |
| *the construction of* | 100 | 64 | noun phrase + of | procedure bundle | 14 | 7 | 26 | 33 | 16 | 4 |
| *this dissertation is* | 99 | 86 | pronoun phrase/noun phrase + be | structuring signal | 7 | 49 | 13 | 8 | 11 | 11 |
| *the field of* | 96 | 74 | noun phrase + of | framing signal | 44 | 5 | 13 | 14 | 19 | 1 |
| *is based on* | 95 | 77 | passive verb + prepositional phrase | framing signal | 19 | 1 | 34 | 33 | 7 | 1 |
| *the framework of* | 94 | 83 | noun phrase + of | description bundle | 15 | 25 | 31 | 9 | 12 | 2 |
| *related to the* | 94 | 78 | passive verb + prepositional phrase | relationship bundle | 13 | 6 | 15 | 55 | 3 | 2 |
| *the nature of* | 93 | 69 | noun phrase + of | description bundle | 30 | 11 | 16 | 22 | 13 | 1 |
| *the degree of* | 93 | 50 | noun phrase + of | description bundle | 6 | 5 | 18 | 60 | 4 | 0 |
| *such as the* | 93 | 81 | other expressions | transition signal | 22 | 1 | 14 | 46 | 10 | 0 |
| *there is a* | 91 | 72 | pronoun phrase/noun phrase + be | causative signal | 24 | 0 | 5 | 52 | 8 | 2 |
| *similarities and differences* | 91 | 49 | other noun phrase | relationship bundle | 8 | 26 | 20 | 25 | 9 | 3 |
| *the concept of* | 90 | 61 | noun phrase + of | description bundle | 21 | 4 | 18 | 27 | 16 | 4 |
| *teaching and learning* | 90 | 59 | other noun phrase | topic bundle | 20 | 5 | 7 | 11 | 47 | 0 |
| *it is found* | 90 | 56 | anticipatory it + verb/adjective phrase | inferential signal | 3 | 1 | 3 | 82 | 1 | 0 |
| *in the Chinese* | 89 | 54 | other prepositional phrase | topic bundle | 15 | 15 | 9 | 33 | 17 | 0 |
| *the number of* | 88 | 65 | noun phrase + of | description bundle | 5 | 0 | 14 | 62 | 3 | 4 |
| *differences in the* | 87 | 56 | noun phrase + other post modifier | relationship bundle | 6 | 15 | 11 | 46 | 9 | 0 |
| *that is the* | 85 | 51 | pronoun phrase/noun phrase + be | transition signal | 10 | 2 | 16 | 49 | 5 | 3 |
| *the fact that* | 84 | 68 | noun phrase + other post modifier | description bundle | 12 | 2 | 3 | 62 | 5 | 0 |
| *there is no* | 84 | 49 | pronoun phrase/noun phrase + be | causative signal | 15 | 3 | 10 | 45 | 11 | 0 |
| *the purpose of* | 81 | 72 | noun phrase + of | objective signal | 7 | 24 | 20 | 19 | 5 | 6 |
| *the theoretical framework* | 80 | 64 | other noun phrase | description bundle | 8 | 15 | 37 | 9 | 9 | 2 |
| *findings of the* | 80 | 73 | noun phrase + of | causative signal | 0 | 0 | 5 | 41 | 31 | 3 |
| *features of the* | 80 | 55 | noun phrase + of | description bundle | 7 | 11 | 22 | 28 | 10 | 2 |
| *the theory of* | 79 | 57 | noun phrase + of | description bundle | 18 | 7 | 20 | 18 | 14 | 2 |
| *as a result* | 79 | 66 | other prepositional phrase | causative signal | 20 | 3 | 11 | 41 | 4 | 0 |
| *this study is* | 78 | 66 | pronoun phrase/noun phrase + be | structuring signal | 1 | 30 | 18 | 7 | 21 | 1 |
| *focuses on the* | 78 | 69 | other verb phrase fragment | procedure bundle | 12 | 27 | 19 | 10 | 7 | 3 |
| *the acquisition of* | 77 | 31 | noun phrase + of | causative signal | 15 | 16 | 4 | 33 | 8 | 1 |
| *are as follows* | 77 | 60 | other expressions | structuring signal | 1 | 11 | 4 | 56 | 5 | 0 |
| *of this dissertation* | 76 | 67 | other prepositional phrase | structuring signal | 2 | 22 | 16 | 8 | 16 | 12 |
| *with regard to* | 74 | 65 | other prepositional phrase | framing signal | 14 | 3 | 16 | 34 | 6 | 1 |
| *the effect of* | 74 | 41 | noun phrase + of | causative signal | 12 | 15 | 14 | 28 | 5 | 0 |
| *in other words* | 74 | 66 | other prepositional phrase | transition signal | 11 | 5 | 7 | 45 | 6 | 0 |
| *the findings of* | 73 | 66 | noun phrase + of | causative signal | 2 | 1 | 7 | 19 | 43 | 1 |
| *in which the* | 73 | 65 | other prepositional phrase | framing signal | 23 | 4 | 14 | 27 | 4 | 1 |
| *in the field* | 72 | 58 | other prepositional phrase | framing signal | 40 | 4 | 9 | 8 | 8 | 3 |
| *by means of* | 72 | 62 | prepositional phrase + of | framing signal | 12 | 5 | 19 | 29 | 7 | 0 |
| *and so on* | 72 | 47 | other expressions | transition signal | 14 | 3 | 12 | 40 | 2 | 1 |
| *to investigate the* | 71 | 62 | to-clause | objective signal | 12 | 28 | 23 | 2 | 6 | 0 |
| *three types of* | 71 | 44 | noun phrase + of | quantification bundle | 5 | 5 | 15 | 43 | 2 | 1 |
| *meaning of the* | 71 | 39 | noun phrase + of | description bundle | 10 | 3 | 12 | 41 | 3 | 2 |
| *in the use* | 71 | 29 | other prepositional phrase | framing signal | 3 | 5 | 7 | 50 | 6 | 0 |
| *on the one hand* | 69 | 62 | other prepositional phrase | transition signal | 19 | 1 | 11 | 22 | 15 | 1 |
| *that it is* | 69 | 59 | pronoun phrase/noun phrase + be | other bundle | 13 | 2 | 11 | 30 | 13 | 0 |
| *a kind of* | 69 | 46 | noun phrase + of | quantification bundle | 23 | 0 | 7 | 35 | 3 | 1 |
| *to examine the* | 68 | 52 | to-clause | objective signal | 5 | 21 | 27 | 11 | 4 | 0 |
| *the context of* | 68 | 43 | noun phrase + of | framing signal | 11 | 9 | 20 | 19 | 8 | 1 |
| *at the same time* | 67 | 59 | other prepositional phrase | location bundle | 10 | 1 | 5 | 35 | 15 | 1 |
| *as for the* | 67 | 49 | adverbial clause fragment | framing signal | 7 | 1 | 13 | 44 | 2 | 0 |
| *the notion of* | 66 | 47 | noun phrase + of | description bundle | 16 | 1 | 22 | 17 | 4 | 6 |
| *in the following* | 66 | 64 | other prepositional phrase | structuring signal | 4 | 5 | 14 | 24 | 19 | 0 |
| *the end of* | 65 | 45 | noun phrase + of | structuring signal | 5 | 1 | 15 | 32 | 12 | 0 |
| *the characteristics of* | 65 | 49 | noun phrase + of | description bundle | 5 | 9 | 19 | 27 | 5 | 0 |
| *of the English* | 65 | 42 | other prepositional phrase | framing signal | 10 | 11 | 14 | 21 | 6 | 3 |
| *of cognitive linguistics* | 65 | 41 | other prepositional phrase | topic bundle | 20 | 13 | 13 | 8 | 10 | 1 |
| *an attempt to* | 65 | 61 | noun phrase + other post modifier | objective signal | 5 | 28 | 16 | 4 | 7 | 5 |
| *with respect to* | 64 | 47 | other prepositional phrase | framing signal | 10 | 4 | 10 | 37 | 0 | 3 |
| *contribute to the* | 64 | 56 | other verb phrase fragment | causative signal | 9 | 8 | 4 | 18 | 25 | 0 |
| *a number of* | 64 | 55 | noun phrase + of | quantification bundle | 18 | 2 | 14 | 16 | 10 | 4 |
| *the two languages* | 63 | 40 | other noun phrase | topic bundle | 8 | 1 | 11 | 35 | 6 | 2 |
| *the principle of* | 63 | 38 | noun phrase + of | description bundle | 11 | 3 | 8 | 35 | 6 | 0 |
| *as a whole* | 63 | 52 | other prepositional phrase | framing signal | 11 | 0 | 11 | 32 | 8 | 1 |
| *different types of* | 62 | 46 | noun phrase + of | quantification bundle | 5 | 6 | 11 | 32 | 7 | 1 |
| *can be used* | 62 | 47 | other verb phrase fragment | procedure bundle | 10 | 5 | 10 | 25 | 11 | 1 |
| *the relation between* | 61 | 43 | noun phrase + other post modifier | relationship bundle | 11 | 6 | 12 | 25 | 5 | 2 |
| *the choice of* | 61 | 44 | noun phrase + of | description bundle | 10 | 2 | 10 | 28 | 7 | 4 |
| *refers to the* | 61 | 41 | other verb phrase fragment | relationship bundle | 28 | 1 | 7 | 24 | 1 | 0 |
| *of the research* | 61 | 51 | other prepositional phrase | structuring signal | 4 | 8 | 14 | 8 | 14 | 13 |
| *the translation of* | 60 | 21 | noun phrase + of | description bundle | 3 | 3 | 16 | 28 | 7 | 3 |
| *the level of* | 60 | 30 | noun phrase + of | description bundle | 6 | 1 | 4 | 45 | 2 | 2 |
| *to make a* | 59 | 56 | to-clause | objective signal | 13 | 19 | 17 | 5 | 5 | 0 |
| *which can be* | 58 | 52 | pronoun phrase/noun phrase + be | procedure bundle | 13 | 0 | 5 | 33 | 7 | 0 |
| *the impact of* | 58 | 24 | noun phrase + of | causative signal | 9 | 7 | 9 | 24 | 6 | 3 |
| *found that the* | 58 | 45 | verb phrase + that-clause | inferential signal | 2 | 0 | 2 | 50 | 4 | 0 |
| *to find out* | 57 | 43 | to-clause | objective signal | 6 | 18 | 22 | 6 | 4 | 1 |
| *the interpretation of* | 57 | 37 | noun phrase + of | procedure bundle | 9 | 3 | 9 | 30 | 6 | 0 |
| *the interaction between* | 57 | 48 | noun phrase + other post modifier | relationship bundle | 6 | 2 | 8 | 34 | 6 | 1 |
| *the combination of* | 57 | 46 | noun phrase + of | quantification bundle | 7 | 4 | 11 | 23 | 12 | 0 |
| *second language acquisition* | 57 | 44 | other noun phrase | topic bundle | 31 | 1 | 10 | 2 | 13 | 0 |
| *present study has* | 57 | 39 | subject + verb phrase+(that-clause) | structuring signal | 0 | 5 | 4 | 8 | 40 | 0 |
| *the function of* | 56 | 35 | noun phrase + of | description bundle | 5 | 1 | 8 | 34 | 8 | 0 |
| *results of the* | 56 | 45 | noun phrase + of | causative signal | 0 | 0 | 8 | 35 | 12 | 1 |
| *present study is* | 56 | 48 | pronoun phrase/noun phrase + be | structuring signal | 3 | 21 | 16 | 2 | 13 | 1 |
| *involved in the* | 56 | 48 | passive verb + prepositional phrase | procedure bundle | 11 | 5 | 13 | 23 | 3 | 1 |
| *to be more* | 55 | 47 | to-clause | objective signal | 7 | 6 | 6 | 31 | 5 | 0 |
| *to account for* | 55 | 45 | to-clause | objective signal | 18 | 2 | 13 | 11 | 11 | 0 |
| *the previous studies* | 55 | 46 | other noun phrase | description bundle | 20 | 3 | 10 | 11 | 4 | 7 |
| *the form of* | 55 | 49 | noun phrase + of | description bundle | 8 | 3 | 11 | 30 | 3 | 0 |
| *of the most* | 55 | 49 | other prepositional phrase | stance features | 36 | 1 | 6 | 11 | 1 | 0 |
| *a variety of* | 55 | 49 | noun phrase + of | quantification bundle | 19 | 2 | 9 | 19 | 4 | 2 |
| *a set of* | 55 | 41 | noun phrase + of | quantification bundle | 12 | 4 | 15 | 21 | 2 | 1 |
| *within the framework* | 54 | 48 | other prepositional phrase | framing signal | 11 | 17 | 16 | 6 | 4 | 0 |
| *used in the* | 54 | 49 | passive verb + prepositional phrase | procedure bundle | 8 | 1 | 22 | 16 | 4 | 3 |
| *with each other* | 53 | 43 | other prepositional phrase | relationship bundle | 13 | 5 | 10 | 19 | 6 | 0 |
| *quantitative and qualitative* | 53 | 46 | other expressions | procedure bundle | 0 | 3 | 33 | 11 | 6 | 0 |
| *native speakers of* | 53 | 26 | noun phrase + of | topic bundle | 2 | 4 | 26 | 15 | 2 | 4 |
| *focus on the* | 53 | 48 | other verb phrase fragment | procedure bundle | 22 | 5 | 10 | 8 | 5 | 3 |
| *be divided into* | 53 | 36 | passive verb + prepositional phrase | procedure bundle | 9 | 1 | 5 | 35 | 0 | 3 |
| *attention to the* | 53 | 47 | noun phrase + other post modifier | engagement feature | 22 | 2 | 3 | 13 | 13 | 0 |
| *the importance of* | 52 | 52 | noun phrase + of | stance feature | 21 | 0 | 7 | 13 | 10 | 1 |
| *the features of* | 52 | 42 | noun phrase + of | description bundle | 9 | 10 | 9 | 20 | 4 | 0 |
| *the differences in* | 52 | 33 | noun phrase + other post modifier | relationship bundle | 5 | 9 | 11 | 15 | 11 | 1 |
| *that of the* | 52 | 38 | noun phrase + of | framing signal | 3 | 2 | 7 | 38 | 2 | 0 |
| *foreign language teaching* | 52 | 32 | other noun phrase | topic bundle | 10 | 3 | 4 | 8 | 21 | 6 |
| *different levels of* | 52 | 37 | noun phrase + of | description bundle | 7 | 7 | 14 | 23 | 1 | 0 |
| *we find that* | 51 | 28 | subject + verb phrase+(that-clause) | inferential signal | 6 | 1 | 4 | 38 | 2 | 0 |
| *the result of* | 51 | 41 | noun phrase + of | causative signal | 5 | 1 | 7 | 35 | 2 | 1 |
| *of the dissertation* | 51 | 42 | other prepositional phrase | structuring signal | 3 | 2 | 8 | 9 | 12 | 17 |
| *of language use* | 51 | 38 | other prepositional phrase | framing signal | 16 | 5 | 7 | 12 | 11 | 0 |
| *light on the* | 51 | 48 | noun phrase + other post modifier | causative signal | 1 | 0 | 3 | 6 | 41 | 0 |
| *description of the* | 51 | 36 | noun phrase + of | description bundle | 7 | 2 | 12 | 19 | 10 | 1 |
| *a foreign language* | 51 | 38 | other noun phrase | topic bundle | 17 | 15 | 7 | 8 | 4 | 0 |
| *this study has* | 50 | 41 | subject + verb phrase+(that-clause) | structuring signal | 0 | 2 | 2 | 15 | 31 | 0 |
| *in the context* | 50 | 41 | other prepositional phrase | framing signal | 12 | 4 | 11 | 18 | 4 | 1 |
| *in relation to* | 50 | 32 | other prepositional phrase | relationship bundle | 7 | 3 | 6 | 22 | 11 | 1 |
| *the most important* | 49 | 44 | other expressions | stance feature | 22 | 0 | 3 | 20 | 4 | 0 |
| *the major findings* | 49 | 47 | other noun phrase | causative signal | 0 | 0 | 3 | 38 | 1 | 7 |
| *most of the* | 49 | 40 | noun phrase + of | quantification bundle | 21 | 0 | 6 | 20 | 2 | 0 |
| *is that the* | 49 | 42 | be + noun/adjective phrase | other bundle | 17 | 0 | 2 | 26 | 4 | 0 |
| *use of the* | 48 | 35 | noun phrase + of | description bundle | 4 | 4 | 3 | 32 | 5 | 0 |
| *theoretical framework of* | 48 | 43 | noun phrase + of | description bundle | 8 | 16 | 17 | 2 | 4 | 1 |
| *the target language* | 48 | 32 | other noun phrase | topic bundle | 7 | 1 | 7 | 25 | 8 | 0 |
| *the present dissertation* | 48 | 39 | other noun phrase | structuring signal | 3 | 26 | 7 | 1 | 6 | 5 |
| *the functions of* | 48 | 33 | noun phrase + of | description bundle | 9 | 2 | 11 | 20 | 5 | 1 |
| *better understanding of* | 48 | 47 | noun phrase + of | stance feature | 7 | 3 | 3 | 8 | 27 | 0 |
| *to be the* | 47 | 40 | to-clause | objective signal | 10 | 1 | 4 | 31 | 1 | 0 |
| *role in the* | 47 | 43 | noun phrase + other post modifier | causative signal | 13 | 0 | 5 | 25 | 4 | 0 |
| *of the target* | 47 | 29 | other prepositional phrase | framing signal | 1 | 0 | 14 | 27 | 5 | 0 |
| *of all the* | 47 | 34 | other prepositional phrase | description bundle | 11 | 0 | 7 | 27 | 1 | 1 |
| *is intended to* | 47 | 40 | (passive) verb phrase + to-clause | objective signal | 6 | 28 | 5 | 3 | 3 | 2 |
| *is composed of* | 47 | 35 | passive verb + prepositional phrase | description bundle | 3 | 1 | 14 | 18 | 1 | 10 |
| *of the semantic* | 46 | 29 | noun phrase + of | location bundle | 6 | 3 | 9 | 26 | 2 | 0 |
| *determined by the* | 46 | 35 | passive verb + prepositional phrase | procedure bundle | 4 | 2 | 2 | 33 | 5 | 0 |
| *what is the* | 45 | 28 | pronoun phrase/noun phrase + be | other bundle | 1 | 38 | 5 | 1 | 0 | 0 |
| *the part of* | 45 | 35 | noun phrase + of | structuring signal | 11 | 1 | 3 | 22 | 5 | 3 |
| *of the language* | 45 | 39 | other prepositional phrase | framing signal | 12 | 2 | 5 | 23 | 3 | 0 |
| *it is also* | 45 | 41 | anticipatory it + verb/adjective phrase | other bundle | 3 | 0 | 2 | 30 | 9 | 1 |
| *in this research* | 45 | 38 | other prepositional phrase | structuring signal | 4 | 3 | 19 | 11 | 6 | 2 |
| *account of the* | 45 | 36 | noun phrase + of | procedure bundle | 5 | 10 | 11 | 12 | 5 | 2 |
| *a series of* | 45 | 39 | noun phrase + of | quantification bundle | 5 | 3 | 19 | 15 | 3 | 0 |
| *to the study* | 44 | 41 | other prepositional phrase | structuring signal | 9 | 2 | 7 | 6 | 18 | 2 |
| *to reveal the* | 44 | 41 | to-clause | objective signal | 7 | 14 | 11 | 8 | 4 | 0 |
| *to analyze the* | 44 | 40 | to-clause | objective signal | 5 | 7 | 23 | 6 | 3 | 0 |
| *the understanding of* | 44 | 42 | noun phrase + of | description bundle | 5 | 1 | 3 | 10 | 25 | 0 |
| *the application of* | 44 | 41 | noun phrase + of | procedure bundle | 17 | 10 | 6 | 3 | 6 | 2 |
| *the research on* | 44 | 41 | noun phrase + other post modifier | procedure bundle | 3 | 8 | 9 | 11 | 12 | 1 |
| *framework of the* | 44 | 41 | noun phrase + of | description bundle | 5 | 6 | 18 | 8 | 2 | 5 |
| *nature of the* | 43 | 35 | noun phrase + of | description bundle | 9 | 5 | 8 | 18 | 3 | 0 |
| *point of view* | 43 | 33 | noun phrase + of | description bundle | 10 | 1 | 4 | 2 | 25 | 1 |
| *our understanding of* | 43 | 39 | noun phrase + of | engagement feature | 8 | 4 | 9 | 18 | 4 | 0 |
| *in the two* | 43 | 36 | other prepositional phrase | framing signal | 6 | 1 | 10 | 23 | 2 | 1 |
| *found to be* | 43 | 35 | (passive) verb phrase + to-clause | inferential signal | 9 | 2 | 4 | 27 | 1 | 0 |
| *based on a* | 43 | 37 | passive verb + prepositional phrase | framing signal | 6 | 5 | 16 | 10 | 6 | 0 |
| *as to the* | 43 | 35 | adverbial clause fragment | framing signal | 8 | 0 | 8 | 23 | 4 | 0 |
| *the structure of* | 42 | 36 | noun phrase + of | description bundle | 9 | 2 | 7 | 17 | 2 | 5 |
| *the scope of* | 42 | 39 | noun phrase + of | description bundle | 7 | 7 | 6 | 8 | 13 | 1 |
| *the relations between* | 42 | 22 | noun phrase + other post modifier | relationship bundle | 5 | 10 | 3 | 20 | 3 | 1 |
| *the English language* | 42 | 24 | other noun phrase | topic bundle | 15 | 2 | 1 | 19 | 5 | 0 |
| *more and more* | 42 | 37 | other expressions | quantification bundle | 25 | 0 | 1 | 14 | 2 | 0 |
| *it has been* | 42 | 33 | pronoun phrase/noun phrase + be | causative signal | 13 | 0 | 1 | 25 | 2 | 1 |
| *is the most* | 42 | 38 | be + noun/adjective phrase | stance feature | 12 | 0 | 5 | 25 | 0 | 0 |
| *is one of* | 42 | 36 | be + noun/adjective phrase | quantification bundle | 28 | 0 | 3 | 8 | 3 | 0 |
| *in the same* | 42 | 35 | other prepositional phrase | framing signal | 7 | 2 | 7 | 21 | 5 | 0 |
| *development of the* | 42 | 31 | noun phrase + of | description bundle | 4 | 8 | 9 | 15 | 6 | 0 |
| *concerned with the* | 42 | 40 | passive verb + prepositional phrase | relationship bundle | 13 | 7 | 4 | 9 | 3 | 6 |
| *closely related to* | 42 | 36 | passive verb + prepositional phrase | relationship bundle | 11 | 0 | 3 | 22 | 5 | 1 |
| *characteristics of the* | 42 | 37 | noun phrase + of | description bundle | 4 | 3 | 14 | 18 | 3 | 0 |
| *theoretical and practical* | 41 | 38 | other expressions | description bundle | 6 | 3 | 1 | 3 | 27 | 1 |
| *the production of* | 41 | 26 | noun phrase + of | causative signal | 7 | 4 | 4 | 25 | 1 | 0 |
| *of the same* | 41 | 34 | other prepositional phrase | framing signal | 6 | 1 | 5 | 28 | 1 | 0 |
| *is concerned with* | 41 | 35 | passive verb + prepositional phrase | relationship bundle | 11 | 7 | 8 | 5 | 2 | 8 |
| *in addition to* | 41 | 38 | other prepositional phrase | transition signal | 6 | 0 | 7 | 19 | 8 | 1 |
| *home and abroad* | 41 | 37 | adverbial clause fragment | location bundle | 19 | 3 | 8 | 2 | 4 | 5 |
| *acquisition of English* | 41 | 18 | noun phrase + of | topic bundle | 7 | 10 | 5 | 14 | 5 | 0 |
| *a process of* | 41 | 33 | noun phrase + of | description bundle | 11 | 0 | 6 | 16 | 7 | 1 |
| *the effectiveness of* | 40 | 26 | noun phrase + of | description bundle | 4 | 5 | 4 | 13 | 14 | 0 |
| *that there is* | 40 | 36 | pronoun phrase/noun phrase + be | causative signal | 7 | 0 | 2 | 25 | 5 | 1 |
| *speakers of English* | 40 | 24 | other noun phrase | topic bundle | 2 | 4 | 17 | 11 | 2 | 4 |
| *show that the* | 40 | 35 | verb phrase + that-clause | inferential signal | 0 | 0 | 5 | 35 | 0 | 0 |
| *qualitative and quantitative* | 40 | 37 | other expressions | procedure bundle | 1 | 1 | 25 | 6 | 7 | 0 |
| *of the text* | 40 | 25 | other prepositional phrase | structuring signal | 7 | 1 | 13 | 16 | 2 | 1 |
| *lies in the* | 40 | 40 | other verb phrase fragment | procedure bundle | 5 | 0 | 5 | 16 | 14 | 0 |
| *it is not* | 40 | 40 | anticipatory it + verb/adjective phrase | other bundle | 8 | 2 | 3 | 20 | 7 | 0 |
| *it is argued* | 40 | 28 | anticipatory it + verb/adjective phrase | inferential signal | 4 | 1 | 3 | 23 | 7 | 2 |
| *in the first* | 40 | 35 | other prepositional phrase | structuring signal | 7 | 0 | 16 | 15 | 1 | 1 |
| *can also be* | 40 | 37 | other verb phrase fragment | procedure bundle | 5 | 1 | 2 | 30 | 2 | 0 |
| *to provide a* | 39 | 38 | to-clause | objective signal | 8 | 12 | 4 | 6 | 7 | 2 |
| *the realization of* | 39 | 29 | noun phrase + of | procedure bundle | 4 | 6 | 5 | 19 | 5 | 0 |
| *the mechanism of* | 39 | 32 | noun phrase + of | description bundle | 8 | 4 | 5 | 13 | 8 | 1 |
| *the establishment of* | 39 | 32 | noun phrase + of | procedure bundle | 5 | 1 | 8 | 15 | 8 | 2 |
| *is not only* | 39 | 37 | be + noun/adjective phrase | transition signal | 18 | 0 | 3 | 17 | 1 | 0 |
| *found in the* | 39 | 38 | passive verb + prepositional phrase | inferential signal | 7 | 0 | 1 | 29 | 2 | 0 |
| *for the purpose* | 39 | 35 | other prepositional phrase | objective signal | 6 | 6 | 11 | 10 | 3 | 3 |
| *first of all* | 39 | 33 | other expressions | structuring signal | 6 | 1 | 14 | 16 | 2 | 0 |
| *differences and similarities* | 39 | 22 | other noun phrase | relationship bundle | 1 | 9 | 14 | 7 | 5 | 3 |
| *at the end* | 39 | 28 | other prepositional phrase | structuring signal | 1 | 0 | 6 | 23 | 9 | 0 |
| *syntactic and semantic* | 39 | 33 | other expressions | topic bundle | 10 | 5 | 5 | 16 | 3 | 0 |
| *in line with* | 38 | 35 | other prepositional phrase | relationship bundle | 4 | 3 | 10 | 17 | 4 | 0 |
| *to study the* | 38 | 33 | to-clause | objective signal | 6 | 8 | 12 | 7 | 5 | 0 |
| *the following three* | 38 | 37 | other expressions | structuring signal | 1 | 15 | 8 | 7 | 7 | 0 |
| *that there are* | 38 | 37 | pronoun phrase/noun phrase + be | causative signal | 5 | 0 | 4 | 26 | 3 | 0 |
| *studies have been* | 38 | 28 | pronoun phrase/noun phrase + be | structuring signal | 23 | 0 | 12 | 3 | 0 | 0 |
| *shows that the* | 38 | 32 | verb phrase + that-clause | inferential signal | 1 | 0 | 2 | 33 | 2 | 0 |
| *of this research* | 38 | 33 | other prepositional phrase | structuring signal | 0 | 3 | 6 | 4 | 17 | 8 |
| *of the whole* | 38 | 36 | other prepositional phrase | framing signal | 4 | 1 | 6 | 16 | 1 | 10 |
| *in accordance with* | 38 | 37 | other prepositional phrase | relationship bundle | 4 | 0 | 11 | 16 | 5 | 2 |
| *as one of* | 38 | 35 | prepositional phrase + of | quantification bundle | 21 | 1 | 5 | 5 | 6 | 0 |
| *are used to* | 38 | 30 | (passive) verb phrase + to-clause | procedure bundle | 5 | 2 | 21 | 9 | 1 | 0 |
| *a contrastive study* | 38 | 24 | other noun phrase | description bundle | 5 | 11 | 11 | 3 | 5 | 3 |
| *a combination of* | 38 | 32 | noun phrase + of | quantification bundle | 4 | 0 | 16 | 12 | 6 | 0 |
| *study aims to* | 38 | 33 | subject + verb phrase+(that-clause) | objective signal | 0 | 33 | 4 | 1 | 0 | 0 |
| *study attempts to* | 38 | 30 | subject + verb phrase+(that-clause) | objective signal | 1 | 29 | 3 | 3 | 2 | 0 |
| *the validity of* | 37 | 32 | noun phrase + of | description bundle | 6 | 4 | 10 | 10 | 6 | 1 |
| *with the help of* | 37 | 32 | prepositional phrase + of | framing signal | 6 | 7 | 11 | 7 | 5 | 1 |
| *the exploration of* | 37 | 34 | noun phrase + of | procedure bundle | 10 | 5 | 5 | 3 | 13 | 1 |
| *the current study* | 37 | 27 | other noun phrase | structuring signal | 5 | 8 | 8 | 1 | 10 | 5 |
| *results show that* | 37 | 31 | subject + verb phrase+(that-clause) | inferential signal | 0 | 0 | 3 | 34 | 0 | 0 |
| *of second language* | 37 | 26 | other prepositional phrase | framing signal | 16 | 4 | 8 | 4 | 5 | 0 |
| *learners of English* | 37 | 25 | other noun phrase | topic bundle | 15 | 4 | 10 | 8 | 0 | 0 |
| *in light of* | 37 | 35 | prepositional phrase + of | framing signal | 7 | 7 | 11 | 5 | 5 | 2 |
| *a second language* | 37 | 31 | other noun phrase | topic bundle | 18 | 3 | 1 | 4 | 11 | 0 |
| *compared with the* | 37 | 37 | passive verb + prepositional phrase | relationship bundle | 6 | 1 | 6 | 24 | 0 | 0 |
| *as far as* | 37 | 30 | other prepositional phrase | framing signal | 9 | 0 | 1 | 22 | 5 | 0 |
| *and the other* | 37 | 35 | other expressions | transition signal | 5 | 0 | 12 | 18 | 2 | 0 |
| *in recent years* | 37 | 25 | other prepositional phrase | location bundle | 33 | 1 | 2 | 0 | 1 | 0 |
| *the change of* | 37 | 35 | noun phrase + of | causative signal | 4 | 2 | 5 | 26 | 0 | 0 |
| *important role in* | 37 | 34 | noun phrase + other post modifier | stance feature | 12 | 2 | 0 | 18 | 5 | 0 |
| *be classified into* | 37 | 31 | passive verb + prepositional phrase | procedure bundle | 9 | 1 | 5 | 17 | 4 | 1 |
| *a lot of* | 37 | 30 | noun phrase + of | quantification bundle | 24 | 0 | 3 | 10 | 0 | 0 |
| *semantic features of* | 37 | 22 | noun phrase + of | description bundle | 9 | 6 | 6 | 13 | 1 | 2 |
| *there are some* | 37 | 33 | pronoun phrase/noun phrase + be | causative signal | 7 | 0 | 0 | 0 | 8 | 22 |
| *emphasis on the* | 37 | 31 | noun phrase + other post modifier | engagement feature | 10 | 2 | 10 | 13 | 1 | 1 |
| *the significance of* | 37 | 31 | noun phrase + of | stance feature | 5 | 1 | 5 | 4 | 14 | 8 |
| *dissertation consists of* | 37 | 30 | subject + verb phrase+(that-clause) | structuring signal | 0 | 0 | 3 | 1 | 0 | 33 |

Note. B denotes the move of Background, G denotes Goal, M denotes Methodology, R denotes Result, C denote Conclusion, and S denotes Structure.

Table 2. Frequent AUC lexical bundles

| Lexical bundle | Token | Text | Structure | Function | Token in rhetorical moves | | | | | |
| --- | --- | --- | --- | --- | --- | --- | --- | --- | --- | --- |
|  |  |  |  |  | B | G | M | R | C | S |
| *I argue that* | 188 | 126 | subject + verb phrase+(that-clause) | inferential signal | 0 | 18 | 5 | 160 | 5 | 0 |
| *as well as* | 186 | 142 | other expression | transition signal | 15 | 23 | 54 | 72 | 15 | 7 |
| *in this dissertation* | 105 | 96 | other prepositional phrase | structuring signal | 2 | 67 | 13 | 19 | 4 | 0 |
| *the use of* | 102 | 72 | noun phrase + of | description signal | 8 | 14 | 29 | 44 | 3 | 4 |
| *I show that* | 96 | 77 | subject + verb phrase+(that-clause) | inferential signal | 0 | 0 | 3 | 92 | 1 | 0 |
| *in order to* | 93 | 78 | other prepositional phrase | objective signal | 6 | 12 | 53 | 18 | 3 | 1 |
| *in terms of* | 86 | 69 | prepositional phrase + of | framing signal | 4 | 8 | 17 | 49 | 5 | 3 |
| *the role of* | 78 | 66 | noun phrase + of | causative signal | 10 | 21 | 19 | 14 | 12 | 2 |
| *a number of* | 74 | 63 | noun phrase + of | quantification bundle | 16 | 9 | 12 | 33 | 4 | 0 |
| *this dissertation is* | 66 | 59 | pronoun phrase/noun phrase + be | structuring signal | 0 | 53 | 2 | 6 | 4 | 1 |
| *the results of* | 61 | 48 | noun phrase + of | causative signal | 0 | 4 | 10 | 28 | 10 | 9 |
| *a variety of* | 60 | 52 | noun phrase + of | quantification bundle | 7 | 9 | 18 | 22 | 3 | 1 |
| *the nature of* | 56 | 50 | noun phrase + of | description bundle | 12 | 9 | 12 | 19 | 3 | 1 |
| *the development of* | 56 | 46 | noun phrase + of | description bundle | 8 | 17 | 13 | 13 | 4 | 1 |
| *part of the* | 55 | 45 | noun phrase + of | structuring signal | 6 | 3 | 22 | 19 | 1 | 4 |
| *with respect to* | 54 | 42 | other prepositional phrase | framing signal | 3 | 6 | 11 | 28 | 4 | 2 |
| *this dissertation examines* | 54 | 54 | subject + verb phrase+(that-clause) | structuring signal | 1 | 49 | 4 | 0 | 0 | 0 |
| *in which the* | 53 | 49 | other prepositional phrase | framing signal | 11 | 2 | 17 | 20 | 1 | 2 |
| *this dissertation investigates* | 50 | 50 | subject + verb phrase+(that-clause) | structuring signal | 1 | 49 | 0 | 0 | 0 | 0 |
| *of the dissertation* | 49 | 38 | other prepositional phrase | structuring signal | 0 | 3 | 27 | 10 | 1 | 8 |
| *one of the* | 48 | 41 | noun phrase + of | quantification bundle | 18 | 3 | 9 | 13 | 4 | 1 |
| *to account for* | 47 | 39 | to-clause | objective signal | 2 | 5 | 12 | 25 | 3 | 0 |
| *based on the* | 47 | 44 | passive verb + prepositional phrase | framing signal | 2 | 8 | 18 | 16 | 0 | 3 |
| *the study of* | 45 | 37 | noun phrase + of | description bundle | 8 | 9 | 10 | 9 | 8 | 1 |
| *the relationship between* | 45 | 39 | noun phrase + other post modifier | relationship signal | 4 | 10 | 20 | 8 | 2 | 1 |
| *in addition to* | 45 | 45 | other prepositional phrase | transition signal | 5 | 4 | 11 | 20 | 5 | 0 |
| *the distribution of* | 44 | 32 | noun phrase + of | causative signal | 2 | 3 | 12 | 24 | 1 | 2 |
| *the analysis of* | 43 | 39 | noun phrase + of | procedure bundle | 3 | 4 | 14 | 19 | 3 | 0 |
| *of this dissertation* | 41 | 37 | other prepositional phrase | structuring signal | 0 | 24 | 7 | 4 | 3 | 3 |
| *I propose that* | 40 | 38 | subject + verb phrase+(that-clause) | inferential signal | 0 | 5 | 2 | 32 | 1 | 0 |
| *an analysis of* | 40 | 36 | noun phrase + of | procedure bundle | 1 | 4 | 19 | 15 | 1 | 0 |
| *of the language* | 39 | 33 | other prepositional phrase | framing signal | 5 | 9 | 9 | 10 | 3 | 3 |
| *the semantics of* | 38 | 30 | noun phrase + of | description bundle | 5 | 12 | 7 | 10 | 1 | 3 |
| *in the second* | 38 | 36 | other prepositional phrase | structuring signal | 2 | 1 | 14 | 17 | 0 | 4 |
| *the fact that* | 37 | 33 | noun phrase + other post modifier | description bundle | 10 | 5 | 4 | 17 | 1 | 0 |
| *the effects of* | 37 | 28 | noun phrase + of | causative signal | 9 | 9 | 12 | 7 | 0 | 0 |
| *the effect of* | 37 | 33 | noun phrase + of | causative signal | 3 | 10 | 6 | 17 | 1 | 0 |
| *the course of* | 36 | 32 | noun phrase + of | location bundle | 2 | 6 | 19 | 9 | 0 | 0 |
| *in the literature* | 36 | 31 | other prepositional phrase | framing signal | 10 | 5 | 8 | 12 | 1 | 0 |
| *use of the* | 34 | 27 | noun phrase + of | description bundle | 2 | 0 | 8 | 22 | 1 | 1 |
| *there is a* | 34 | 29 | pronoun phrase/noun phrase + be | causative signal | 10 | 2 | 3 | 16 | 2 | 1 |
| *a series of* | 34 | 33 | noun phrase + of | quantification bundle | 1 | 6 | 16 | 10 | 0 | 1 |
| *a range of* | 34 | 31 | noun phrase + of | quantification bundle | 5 | 4 | 14 | 8 | 3 | 0 |
| *second language acquisition* | 33 | 27 | other noun phrase | topic bundle | 17 | 5 | 3 | 4 | 4 | 0 |
| *in this study* | 33 | 30 | other prepositional phrase | structuring signal | 1 | 5 | 15 | 10 | 2 | 0 |
| *ways in which* | 32 | 29 | noun phrase + other post modifier | framing signal | 1 | 8 | 9 | 10 | 3 | 1 |
| *our understanding of* | 32 | 32 | noun phrase + of | engagement features | 3 | 10 | 1 | 5 | 13 | 0 |
| *it is argued* | 32 | 24 | anticipatory it + verb/adjective phrase | inferential signal | 2 | 1 | 0 | 29 | 0 | 0 |
| *this dissertation explores* | 31 | 30 | subject + verb phrase+(that-clause) | structuring signal | 0 | 30 | 1 | 0 | 0 | 0 |
| *the present study* | 31 | 30 | other noun phrase | structuring signal | 2 | 16 | 8 | 2 | 1 | 2 |
| *the presence of* | 31 | 27 | noun phrase + of | causative signal | 2 | 0 | 3 | 22 | 2 | 2 |
| *the acquisition of* | 31 | 21 | noun phrase + of | causative signal | 3 | 11 | 8 | 7 | 1 | 1 |
| *the importance of* | 30 | 29 | noun phrase + of | stance feature | 5 | 2 | 2 | 9 | 11 | 1 |
| *properties of the* | 30 | 26 | noun phrase + of | description bundle | 4 | 10 | 3 | 11 | 1 | 1 |
| *nature of the* | 30 | 28 | noun phrase + of | description bundle | 4 | 5 | 5 | 14 | 1 | 1 |
| *results suggest that* | 29 | 27 | subject + verb phrase+(that-clause) | inferential signal | 0 | 0 | 0 | 28 | 1 | 0 |
| *results show that* | 29 | 26 | subject + verb phrase+(that-clause) | inferential signal | 0 | 0 | 0 | 29 | 0 | 0 |
| *on the other* | 29 | 27 | other prepositional phrase | transition signal | 8 | 1 | 1 | 19 | 0 | 0 |
| *of this study* | 29 | 29 | other prepositional phrase | structuring signal | 0 | 13 | 6 | 7 | 3 | 0 |
| *understanding of the* | 28 | 26 | noun phrase + of | description bundle | 0 | 7 | 2 | 6 | 10 | 3 |
| *this dissertation presents* | 28 | 28 | subject + verb phrase+(that-clause) | structuring signal | 0 | 23 | 1 | 2 | 2 | 0 |
| *of the study* | 28 | 19 | other prepositional phrase | structuring signal | 0 | 1 | 12 | 9 | 3 | 3 |
| *in the first* | 28 | 28 | other prepositional phrase | structuring signal | 1 | 1 | 8 | 15 | 0 | 3 |
| *focuses on the* | 28 | 28 | other verb phrase fragment | procedure bundle | 1 | 9 | 12 | 4 | 0 | 2 |
| *account for the* | 28 | 25 | other verb phrase fragment | procedure bundle | 2 | 4 | 4 | 15 | 1 | 2 |
| *at the same time* | 27 | 25 | other prepositional phrase | location bundle | 9 | 2 | 3 | 10 | 3 | 0 |
| *study of the* | 27 | 26 | noun phrase + of | structuring signal | 3 | 8 | 10 | 4 | 2 | 0 |
| *some of the* | 27 | 25 | noun phrase + of | quantification bundle | 3 | 3 | 6 | 12 | 2 | 1 |
| *it is shown* | 27 | 22 | anticipatory it + verb/adjective phrase | inferential signal | 0 | 0 | 0 | 27 | 0 | 0 |
| *the result of* | 26 | 22 | noun phrase + of | causative signal | 2 | 2 | 4 | 16 | 2 | 0 |
| *results of the* | 26 | 18 | noun phrase + of | causative signal | 0 | 0 | 4 | 12 | 4 | 6 |
| *in this thesis* | 26 | 24 | other prepositional phrase | structuring signal | 0 | 13 | 2 | 8 | 3 | 0 |
| *due to the* | 26 | 25 | predicative adjective + to-clause | causative signal | 6 | 4 | 3 | 12 | 1 | 0 |
| *a set of* | 26 | 24 | noun phrase + of | quantification bundle | 4 | 3 | 13 | 5 | 1 | 0 |
| *the hypothesis that* | 25 | 24 | noun phrase + other post modifier | description signal | 2 | 6 | 10 | 6 | 1 | 0 |
| *can be used* | 25 | 23 | other verb phrase fragment | procedure signal | 0 | 0 | 4 | 15 | 6 | 0 |
| *are shown to* | 25 | 18 | (passive) verb phrase + to-clause | inferential signal | 1 | 0 | 2 | 22 | 0 | 0 |
| *on the basis of* | 24 | 24 | prepositional phrase + of | framing signal | 3 | 2 | 2 | 14 | 3 | 0 |
| *the process of* | 24 | 21 | noun phrase + of | location bundle | 5 | 3 | 4 | 9 | 2 | 1 |
| *the interaction between* | 24 | 20 | noun phrase + other post modifier | relationship signal | 3 | 2 | 4 | 13 | 2 | 0 |
| *in relation to* | 24 | 21 | other prepositional phrase | relationship signal | 1 | 5 | 7 | 9 | 1 | 1 |
| *between the two* | 24 | 23 | other prepositional phrase | relationship signal | 6 | 1 | 5 | 11 | 1 | 0 |
| *to investigate the* | 23 | 22 | to-clause | objective signal | 1 | 10 | 11 | 0 | 1 | 0 |
| *the production of* | 23 | 19 | noun phrase + of | causative signal | 2 | 3 | 9 | 6 | 3 | 0 |
| *the goal of* | 23 | 21 | noun phrase + of | objective signal | 0 | 16 | 7 | 0 | 0 | 0 |
| *the field of* | 23 | 22 | noun phrase + of | framing signal | 13 | 1 | 0 | 5 | 3 | 1 |
| *the extent to which* | 23 | 22 | noun phrase + other post modifier | description bundle | 3 | 4 | 8 | 5 | 3 | 0 |
| *that there is* | 23 | 21 | pronoun phrase/noun phrase + be | causative signal | 5 | 3 | 0 | 14 | 1 | 0 |
| *such as the* | 23 | 22 | other expressions | transition signal | 3 | 1 | 4 | 13 | 2 | 0 |
| *structure of the* | 23 | 21 | noun phrase + of | description bundle | 3 | 4 | 5 | 11 | 0 | 0 |
| *results indicate that* | 23 | 23 | subject + verb phrase+(that-clause) | inferential signal | 0 | 0 | 0 | 22 | 1 | 0 |
| *more likely to* | 23 | 19 | predicative adjective + to-clause | stance feature | 3 | 1 | 2 | 17 | 0 | 0 |
| *goal of this* | 23 | 23 | noun phrase + of | objective signal | 0 | 20 | 1 | 1 | 1 | 0 |
| *associated with the* | 23 | 20 | passive verb + prepositional phrase | relationship signal | 1 | 5 | 7 | 9 | 0 | 1 |
| *as a result* | 23 | 21 | other prepositional phrase | causative signal | 5 | 3 | 3 | 12 | 0 | 0 |
| *account of the* | 23 | 23 | noun phrase + of | procedure bundle | 1 | 6 | 2 | 11 | 2 | 1 |
| *to show that* | 22 | 21 | to-clause | objective signal | 0 | 4 | 7 | 10 | 1 | 0 |
| *the structure of* | 22 | 21 | noun phrase + of | description bundle | 6 | 4 | 5 | 7 | 0 | 0 |
| *the notion of* | 22 | 20 | noun phrase + of | description bundle | 2 | 4 | 6 | 7 | 2 | 1 |
| *the level of* | 22 | 21 | noun phrase + of | description bundle | 0 | 3 | 3 | 14 | 2 | 0 |
| *the interpretation of* | 22 | 16 | noun phrase + of | procedure bundle | 1 | 9 | 3 | 5 | 3 | 1 |
| *the context of* | 22 | 20 | noun phrase + of | framing signal | 3 | 5 | 7 | 6 | 1 | 0 |
| *that have been* | 22 | 21 | pronoun phrase/noun phrase + be | causative signal | 3 | 3 | 6 | 9 | 1 | 0 |
| *of the two* | 22 | 21 | other prepositional phrase | framing signal | 0 | 2 | 9 | 9 | 1 | 1 |
| *in the syntax* | 22 | 14 | other prepositional phrase | framing signal | 5 | 3 | 1 | 12 | 1 | 0 |
| *in the language* | 22 | 20 | other prepositional phrase | framing signal | 3 | 3 | 3 | 10 | 1 | 2 |
| *I demonstrate that* | 22 | 20 | subject + verb phrase+(that-clause) | inferential signal | 0 | 3 | 0 | 19 | 0 | 0 |
| *a model of* | 22 | 21 | noun phrase + of | description bundle | 0 | 7 | 1 | 11 | 3 | 0 |
| *syntactic and semantic* | 22 | 17 | other expressions | topic bundle | 3 | 3 | 8 | 7 | 1 | 0 |
| *in the case of* | 21 | 19 | prepositional phrase + of | framing signal | 4 | 0 | 7 | 10 | 0 | 0 |
| *to examine the* | 21 | 21 | to-clause | objective signal | 3 | 9 | 8 | 1 | 0 | 0 |
| *there is no* | 21 | 20 | pronoun phrase/noun phrase + be | causative signal | 0 | 0 | 2 | 19 | 0 | 0 |
| *the theory of* | 21 | 19 | noun phrase + of | description bundle | 4 | 3 | 5 | 6 | 1 | 2 |
| *the question of* | 21 | 21 | noun phrase + of | description bundle | 6 | 3 | 7 | 2 | 1 | 2 |
| *the purpose of* | 21 | 19 | noun phrase + of | objective signal | 0 | 18 | 2 | 1 | 0 | 0 |
| *shown to be* | 21 | 16 | (passive) verb phrase + to-clause | inferential signal | 1 | 0 | 1 | 19 | 0 | 0 |
| *related to the* | 21 | 15 | passive verb + prepositional phrase | relationship signal | 1 | 0 | 4 | 15 | 0 | 1 |
| *in the study* | 21 | 19 | other prepositional phrase | structuring signal | 4 | 1 | 9 | 5 | 2 | 0 |
| *in favor of* | 21 | 19 | prepositional phrase + of | relationship signal | 2 | 1 | 5 | 12 | 1 | 0 |
| *evidence for the* | 21 | 18 | noun phrase + other post modifier | causative signal | 3 | 1 | 2 | 10 | 3 | 2 |
| *attention to the* | 21 | 19 | noun phrase + other post modifier | engagement feature | 7 | 2 | 2 | 6 | 3 | 1 |
| *two types of* | 20 | 17 | noun phrase + of | quantification bundle | 2 | 1 | 3 | 13 | 1 | 0 |
| *the speech of* | 20 | 18 | noun phrase + of | description bundle | 6 | 5 | 4 | 5 | 0 | 0 |
| *the dissertation is* | 20 | 18 | pronoun phrase/noun phrase + be | structuring signal | 0 | 5 | 3 | 5 | 2 | 5 |
| *that can be* | 20 | 19 | pronoun phrase/noun phrase + be | procedure bundle | 4 | 1 | 4 | 8 | 3 | 0 |
| *many of the* | 20 | 20 | noun phrase + of | quantification bundle | 2 | 2 | 3 | 11 | 2 | 0 |
| *is not a* | 20 | 20 | be + noun/adjective phrase | other bundle | 1 | 2 | 1 | 12 | 3 | 1 |
| *in the same* | 20 | 20 | other prepositional phrase | framing signal | 5 | 1 | 4 | 8 | 1 | 1 |
| *in natural language* | 20 | 18 | other prepositional phrase | framing signal | 3 | 5 | 4 | 7 | 1 | 0 |
| *I propose a* | 20 | 19 | subject + verb phrase+(that-clause) | inferential signal | 0 | 5 | 3 | 11 | 1 | 0 |
| *found to be* | 20 | 17 | (passive) verb phrase + to-clause | inferential signal | 2 | 0 | 1 | 17 | 0 | 0 |
| *differences in the* | 20 | 16 | noun phrase + other post modifier | relationship signal | 2 | 3 | 1 | 13 | 0 | 1 |
| *as opposed to* | 20 | 19 | adverbial clause fragment | relationship signal | 1 | 2 | 8 | 9 | 0 | 0 |
| *whether or not* | 19 | 16 | other expressions | other bundle | 5 | 2 | 4 | 5 | 2 | 1 |
| *variation in the* | 19 | 19 | noun phrase + other post modifier | causative signal | 3 | 4 | 3 | 9 | 0 | 0 |
| *this study is* | 19 | 19 | pronoun phrase/noun phrase + be | structuring signal | 1 | 9 | 4 | 3 | 1 | 1 |
| *the influence of* | 19 | 17 | noun phrase + of | causative signal | 4 | 3 | 4 | 8 | 0 | 0 |
| *the ability to* | 19 | 16 | noun phrase + other post modifier | description bundle | 7 | 1 | 3 | 7 | 1 | 0 |
| *of the verb* | 19 | 14 | other prepositional phrase | framing signal | 1 | 0 | 6 | 11 | 0 | 1 |
| *of the same* | 19 | 16 | other prepositional phrase | framing signal | 4 | 1 | 2 | 12 | 0 | 0 |
| *it has been* | 19 | 18 | anticipatory it + verb/adjective phrase | causative signal | 14 | 0 | 1 | 4 | 0 | 0 |
| *I focus on* | 19 | 17 | subject + verb phrase+(that-clause) | procedure bundle | 0 | 5 | 13 | 1 | 0 | 0 |
| *I examine the* | 19 | 18 | subject + verb phrase+(that-clause) | procedure bundle | 1 | 5 | 11 | 2 | 0 | 0 |
| *found in the* | 19 | 17 | passive verb + prepositional phrase | causative signal | 1 | 0 | 6 | 8 | 2 | 2 |
| *each of the* | 19 | 19 | noun phrase + of | quantification bundle | 2 | 1 | 9 | 6 | 0 | 1 |
| *discussion of the* | 19 | 15 | noun phrase + of | procedure bundle | 1 | 1 | 6 | 4 | 4 | 3 |
| *as part of* | 19 | 19 | prepositional phrase + of | framing signal | 2 | 1 | 5 | 10 | 1 | 0 |
| *a theory of* | 19 | 19 | noun phrase + of | description bundle | 1 | 2 | 3 | 6 | 5 | 2 |
| *with regard to* | 18 | 16 | other prepositional phrase | framing signal | 2 | 0 | 3 | 11 | 1 | 1 |
| *the majority of* | 18 | 18 | noun phrase + of | quantification bundle | 4 | 2 | 2 | 9 | 1 | 0 |
| *the interaction of* | 18 | 16 | noun phrase + of | procedure bundle | 1 | 3 | 5 | 6 | 2 | 1 |
| *the existence of* | 18 | 15 | noun phrase + of | causative signal | 2 | 2 | 0 | 13 | 1 | 0 |
| *the absence of* | 18 | 18 | noun phrase + of | causative signal | 1 | 2 | 2 | 11 | 2 | 0 |
| *of the grammar* | 18 | 15 | other prepositional phrase | framing signal | 4 | 5 | 2 | 2 | 4 | 1 |
| *of the data* | 18 | 17 | other prepositional phrase | framing signal | 0 | 1 | 7 | 7 | 0 | 3 |
| *is based on* | 18 | 18 | passive verb + prepositional phrase | framing signal | 2 | 0 | 11 | 5 | 0 | 0 |
| *in the context* | 18 | 18 | other prepositional phrase | framing signal | 1 | 5 | 6 | 5 | 1 | 0 |
| *in light of* | 18 | 18 | prepositional phrase + of | framing signal | 1 | 2 | 3 | 8 | 3 | 1 |
| *implications for the* | 18 | 18 | noun phrase + other post modifier | causative signal | 1 | 1 | 1 | 3 | 10 | 2 |
| *I argue for* | 18 | 17 | subject + verb phrase+(that-clause) | inferential signal | 0 | 0 | 1 | 16 | 1 | 0 |
| *contribute to the* | 18 | 17 | other verb phrase fragment | causative signal | 1 | 0 | 3 | 8 | 6 | 0 |
| *and that the* | 18 | 18 | other expressions | transition signal | 0 | 1 | 0 | 17 | 0 | 0 |
| *a study of* | 18 | 16 | noun phrase + of | procedure bundle | 1 | 8 | 5 | 4 | 0 | 0 |
| *a discussion of* | 18 | 15 | noun phrase + of | procedure bundle | 0 | 0 | 7 | 4 | 3 | 4 |
| *that is the* | 17 | 14 | pronoun phrase/noun phrase + be | transition signal | 3 | 4 | 1 | 8 | 1 | 0 |
| *the range of* | 17 | 17 | noun phrase + of | description bundle | 2 | 2 | 4 | 8 | 1 | 0 |
| *the history of* | 17 | 15 | noun phrase + of | description bundle | 4 | 2 | 2 | 5 | 1 | 3 |
| *the form of* | 17 | 16 | noun phrase + of | description bundle | 3 | 2 | 4 | 8 | 0 | 0 |
| *that it is* | 17 | 17 | pronoun phrase/noun phrase + be | other bundle | 5 | 0 | 1 | 10 | 1 | 0 |
| *be used to* | 17 | 16 | (passive) verb phrase + to-clause | procedure bundle | 0 | 0 | 3 | 8 | 6 | 0 |
| *are consistent with* | 17 | 15 | be + noun/adjective phrase | relationship signal | 0 | 0 | 1 | 14 | 2 | 0 |
| *are able to* | 17 | 14 | pronoun phrase/noun phrase + be | structuring signal | 3 | 1 | 5 | 7 | 1 | 0 |
| *a result of* | 17 | 15 | noun phrase + of | causative signal | 1 | 2 | 3 | 10 | 1 | 0 |
| *used in the* | 16 | 16 | passive verb + prepositional phrase | procedure bundle | 1 | 2 | 8 | 4 | 0 | 1 |
| *the type of* | 16 | 16 | noun phrase + of | description bundle | 4 | 0 | 3 | 9 | 0 | 0 |
| *play a role in* | 16 | 15 | other verb phrase fragment | causative signal | 2 | 2 | 0 | 12 | 0 | 0 |
| *of the target* | 16 | 14 | other prepositional phrase | framing signal | 3 | 1 | 3 | 9 | 0 | 0 |
| *for future research* | 16 | 16 | other prepositional phrase | objective signal | 0 | 0 | 0 | 0 | 10 | 6 |
| *a subset of* | 16 | 16 | noun phrase + of | quantification bundle | 2 | 1 | 5 | 7 | 0 | 1 |
| *a second language* | 16 | 14 | other noun phrase | topic bundle | 6 | 3 | 5 | 2 | 0 | 0 |
| *a case study* | 16 | 14 | other noun phrase | description bundle | 2 | 5 | 4 | 4 | 1 | 0 |
| *this thesis is* | 15 | 15 | pronoun phrase/noun phrase + be | structuring signal | 0 | 11 | 1 | 2 | 1 | 0 |
| *the framework of* | 15 | 14 | noun phrase + of | description bundle | 1 | 1 | 7 | 4 | 2 | 0 |
| *the first study* | 15 | 15 | other noun phrase | description bundle | 0 | 0 | 12 | 3 | 0 | 0 |
| *the current study* | 15 | 15 | other noun phrase | structuring signal | 0 | 9 | 4 | 1 | 1 | 0 |
| *of second language* | 15 | 14 | other prepositional phrase | framing signal | 5 | 4 | 4 | 0 | 2 | 0 |
| *is consistent with* | 15 | 14 | be + noun/adjective phrase | relationship signal | 0 | 0 | 0 | 15 | 0 | 0 |
| *in which a* | 15 | 14 | other prepositional phrase | framing signal | 1 | 2 | 5 | 6 | 1 | 0 |
| *I suggest that* | 15 | 14 | subject + verb phrase+(that-clause) | inferential signal | 0 | 2 | 0 | 12 | 1 | 0 |
| *I show how* | 15 | 14 | subject + verb phrase+(that-clause) | inferential signal | 0 | 2 | 2 | 11 | 0 | 0 |
| *examination of the* | 15 | 15 | noun phrase + of | procedure bundle | 2 | 4 | 3 | 3 | 2 | 1 |
| *contributes to the* | 15 | 15 | other verb phrase fragment | causative signal | 2 | 2 | 1 | 1 | 9 | 0 |
| *are used to* | 15 | 14 | (passive) verb phrase + to-clause | procedure bundle | 0 | 1 | 9 | 4 | 1 | 0 |
| *to determine the* | 15 | 14 | to-clause | objective signal | 2 | 4 | 6 | 3 | 0 | 0 |
| *their ability to* | 15 | 14 | noun phrase + other post modifier | description bundle | 2 | 2 | 2 | 8 | 1 | 0 |

Note. B denotes the move of Background, G denotes Goal, M denotes Methodology, R denotes Result, C denote Conclusion, and S denotes Structure.

# Working taxonomies

Table 3 presents the working structural taxonomy.

Table 3. Working structural taxonomy (modified based on Biber et al., 1999, p. 1014-1015)

| Category | Structure | Example LB (raw frequency) |
| --- | --- | --- |
| NP-based | noun phrase + of | *the use of* (102) |
|  | noun phrase with other post modifier | *the relationship between* (45) |
|  | **other noun phrase fragments** | *Second Language Acquisition* (33) |
| PP-based | prepositional phrase + of | *in terms of* (86) |
|  | other prepositional phrase | *in this dissertation* (105) |
| VP-based | be + noun/adjective phrase | *are consistent with* (17) |
|  | passive verb + prepositional phrase | *based on the* (47) |
|  | anticipatory *it* + verb/adjective phrase | *it is argued that* (28) |
|  | verb phrase + *that*-clause | *show that the* (52) |
|  | **subject + verb phrase + (that-clause)** | *I argue that* (187) |
|  | predicative adjective + to-clause | *due to the* (26) |
|  | (passive) verb phrase + to-clause | *are shown to* (25) |
|  | to-clause | *to account for* (47) |
|  | adverbial clause fragments | *as opposed to* (20) |
|  | pronoun/noun phrase + be | *this dissertation is* (66) |
|  | **other verb phrase fragments** | *focuses on the* (27) |
| other expressions | NA | *as well as* (186) |

Note. Bold font denotes newly-added categories. Examples and their raw frequency are from AUC.

Table 4 presents the working functional taxonomy.

Table 4. Working functional taxonomy (modified based on Hyland, 2008a, p. 49)

| Category | Sub-category | Example LB (raw frequency) |
| --- | --- | --- |
| research-oriented | location | *the course of* (36) |
|  | procedure | *analysis of the* (69) |
|  | quantification | *a number of* (74) |
|  | description | *the use of* (102) |
|  | topic | *Second Language Acquisition* (33) |
| text-oriented | transition signal | *as well as* (186) |
|  | **inferential signal** | *I argue that* (187) |
|  | **causative signal** | *the results of* (61) |
|  | structuring signal | *in this dissertation* (105) |
|  | framing signal | *in terms of* (86) |
|  | **relationship signal** | *the relationship between* (45) |
|  | **objective signal** | *in order to* (93) |
| participant-oriented | stance feature | *the importance of* (30) |
|  | engagement feature | *our understanding of* (32) |
| other bundle | NA | *is not a* (20) |

Note. Bold font denotes newly-added categories. Examples and their raw frequency are from AUC.

Table 5 shows the communicative purposes of move-specific bundles in the six rhetorical moves.

Table 5. Communicative purposes of move-specific bundles

| Move | Communicative purpose | Move-specific bundle | |
| --- | --- | --- | --- |
|  |  | AUC | CUC |
| Background | Emphasize research importance | *one of the* | ***one of the***, *of the most*, *is one of*, ***a lot of***, ***more and more***, ***as one of***, ***the most important***, ***is not only***, *the importance of* |
|  | Introduce previous studies | *it has been*, *the field of* | ***the study of***, ***in the field***, ***the field of***, ***studies have been***, ***refers to the***, ***most of the***, *focus on the*, ***the previous studies***, ***the research on***, ***a kind of***, *a variety of*, *in which the*, ***the relationship between***, ***is that the***, *to account for*, *the nature of* |
|  | Specify research topics | *second language acquisition* | *second language acquisition*, *a second language*, *of second language*, ***learners of English***, ***the English language***, ***a foreign language***, ***of cognitive linguistics***, *of language use* |
|  | Specify geographical distribution of study | NA | ***home and abroad*** |
|  | Specify temporal distribution of study | NA | ***in recent years*** |
|  | Reveal research gaps | NA | *attention to the* |
| Goal | Refer to research | ***in this dissertation***, ***this dissertation is***, *of this dissertation*, *the present study*, *this thesis is*, ***in this thesis***, *of this study*, *the current study*, *this study is*, *a study of*, *study of the* | ***the present study***, *this dissertation is*, ***the present dissertation***, ***this study is***, ***present study is***, *of this dissertation*, ***the present research***, ***a contrastive study***, ***of this study***, *the current study*, ***in this study*** |
|  | Present research purposes | ***this dissertation investigates***, ***this dissertation examines***, ***this dissertation presents***, ***this dissertation explores***, ***goal of this***, *the purpose of*, ***the goal of***, *to investigate the*, *to examine the*, *focuses on the* | ***study aims to***, ***study attempts to***, ***to explore the***, ***is intended to***, ***an attempt to***, *to investigate the*, *focuses on the*, *the purpose of*, *to examine the*, ***to make a***, ***to find out***, ***to reveal the***, *to provide a*, ***so as to***, ***to study the***, *in order to*, *is concerned with*, ***concerned with the***, ***to analyze the*** |
|  | Describe research subjects | *the development of*, ***the role of***, *the acquisition of*, *the interpretation of*, ***the semantics of***, ***properties of the***, ***our understanding of***, *the effect of*, ***a model of*** | ***Chinese EFL learners***, *the effects of*, ***similarities and differences***, *the acquisition of*, *the effect of*, ***English and Chinese***, ***Chinese and English***, ***a foreign language***, ***the relationship between***, ***acquisition of English***, ***study of the***, ***the relations between***, ***differences in the***, ***the research on***, *account of the*, ***in the Chinese***, ***differences and similarities***, ***the features of***, *the role of*, ***of the English***, *development of the*, ***the differences in***, *the application of*, ***features of the***, ***the characteristics of*** |
|  | Display research questions | NA | ***what are the***, ***what is the***, ***the following three*** |
|  | Demonstrate research frameworks and perspectives | NA | ***from the perspective of***, ***the framework of***, ***within the framework***, ***theoretical framework of***, ***on the basis of***, ***based on the***, ***the theoretical framework***, ***of cognitive linguistics***, ***with the help of***, *in light of*, ***are as follows***, *the scope of* |
| Methodology | Describe methodology | ***the course of***, *is based on*, *a set of* | ***based on the***, ***in terms of***, ***on the basis of***, ***qualitative and quantitative***, ***is based on***, ***quantitative and qualitative***, *a series of*, *a combination of*, *based on a* |
|  | Present research subjects | *the relationship between* | *native speakers of*, *speakers of English*, *the notion of*, ***differences and similarities***, *the effects of* |
|  | Narrate research acts | *in order to*, ***I focus on***, ***an analysis of***, ***I examine the***, ***a series of***, *are used to* | *in order to*, ***to analyze the***, *are used to*, *to examine the*, *used in the*, ***to find out***, *the analysis of*, ***to explore the***, *to investigate the* |
|  | Refer to dissertations | ***of the dissertation***, ***the first study***, *part of the*, *in this study* | ***the present study***, ***in this research***, *in the first*, *part of the*, ***first of all***, ***in this study***, ***in the study***, *in this dissertation*, ***of the study*** |
|  | Demonstrate research frameworks | NA | ***according to the***, ***the theoretical framework***, ***framework of the***, ***theoretical framework of***, ***the framework of*** |
| Result | Elicit the report of results | ***I argue that***, ***I show that***, ***results show that***, ***results suggest that***, ***it is shown***, ***it is argued***, ***results indicate that***, ***I propose that***, ***are shown to***, ***shown to be***, ***I demonstrate that***, ***I argue for***, *found to be* | ***it is found***, ***found that the***, *results show that*, *show that the*, *shows that the*, ***are as follows***, ***the major findings***, ***we find that***, *found in the* |
|  | Describe findings | *there is no*, *and that the* | ***the number of***, *the level of*, *the use of*, ***in the use***, ***that of the***, ***there is no***, ***the degree of***, ***can also be***, ***determined by the*** |
|  | Discuss results | ***is consistent with*** | *NA* |
| Conclusion | Refer to dissertations | NA | ***the present study***, ***present study has***, ***this study has***, ***of this study***, *of this research*, ***the present research***, ***this study is***, *of the research*, *the current study*, ***present study is***, *of this dissertation*, *of the dissertation*, *in this dissertation*, ***in this study***, ***at the end***, ***of the study*** |
|  | Refer to findings | ***the results of*** | ***the findings of***, ***findings of the***, *the results of*, *results of the* |
|  | Elicit presentations of implications | ***implications for the***, *contributes to the*, *contribute to the* | *light on the*, *contribute to the*, ***in the following***, ***lies in the*** |
|  | Describe implications | *the importance of* | ***theoretical and practical***, ***the significance of*** |
|  | Stress beneficiaries | ***our understanding of***, *understanding of the*, ***the role of***, ***a theory of***, *the study of*, ***of the grammar***, ***discussion of the*** | ***teaching and learning***, ***better understanding of***, *our understanding of*, ***the understanding of***, ***foreign language teaching***, *to the study*, *the development of*, ***the effectiveness of***, ***the exploration of***, *the scope of*, ***the study of***, *a second language*, ***the field of***, *second language acquisition*, ***study of the***, ***in the Chinese***, ***the combination of***, ***of language use***, ***the differences in*** |
|  | Elicit more contents | NA | *at the same time*, *that it is*, *in relation to*, ***on the other*** |
|  | Suggest future directions | *for future research* | *attention to the* |
|  | Propose applications | *be used to*, *can be used* | ***so as to***, *the application of* |
| Structure | Elicit presentations of structures | NA | ***dissertation consists of***, ***is composed of***, *the structure of*, ***framework of the*** |
|  | Refer to dissertations or chapters | ***of the dissertation***, ***the dissertation is***, ***in the second***, *of the study*, ***in the first***, *part of the* | *of the dissertation*, ***of the research***, ***of the whole***, *of this dissertation*, ***of the study***, *of this research*, *this dissertation is*, *in this dissertation*, *the current study*, *part of the*, ***the present dissertation***, ***of this study*** |
|  | Relate to other contents | *for future research*, ***the results of***, *results of the*, ***a discussion of***, *the history of*, *of the data*, ***discussion of the***, *understanding of the* | ***there are some***, ***the significance of***, *is concerned with*, ***the major findings***, *as well as*, ***the previous studies***, ***English and Chinese***, ***concerned with the***, ***foreign language teaching***, ***home and abroad***, *the notion of*, *the purpose of*, *speakers of English*, ***an attempt to***, *native speakers of* |

Note. Bold font denotes BUDs.

# Sources and amounts of sample dissertation abstracts

Table 6 Sources and amounts of sample dissertation abstracts

| Chinese University | Amount | American University | Amount |
| --- | --- | --- | --- |
| Beijing Foreign Studies University | 53 | Georgetown University | 80 |
| Beijing Normal University | 36 | Harvard University | 33 |
| East China Normal University | 28 | Massachusetts Institute of Technology | 47 |
| Fudan University | 70 | New York University | 51 |
| Guangdong University of Foreign Studies | 93 | Stanford University | 63 |
| Nanjing Normal University | 43 | University of California-Berkeley | 57 |
| Nanjing University | 61 | University of California-Los Angeles | 88 |
| Peking University | 34 | University of California-San Diego | 46 |
| Shandong University | 36 | University of Illinois at Urbana-Champaign | 55 |
| Shanghai International Studies University | 98 | University of Massachusetts | 45 |
| Shanghai Jiao Tong University | 101 | University of Chicago | 40 |
| Tsinghua University | 25 | University of Pennsylvania | 61 |
| Zhejiang University | 22 | University of Texas at Austin | 34 |
